# Comprehensive Characterization of Drying Oil Oxidation and Polymerization Using Time-Resolved Infrared Spectroscopy

**DOI:** 10.1021/acs.macromol.4c01164

**Published:** 2024-08-28

**Authors:** Gwen DePolo, Piet Iedema, Kenneth Shull, Joen Hermans

**Affiliations:** † Materials Science and Engineering, Northwestern University, Evanston, Illinois 60208, United States; ‡ Van ‘t Hoff Institute for Molecular Sciences, University of Amsterdam, P.O. Box 94157, Amsterdam 1090 GD, The Netherlands; § Conservation & Restoration, Amsterdam School of Heritage, Memory and Material Culture, University of Amsterdam, P.O. Box 94551, Amsterdam 1090 GN, The Netherlands; ∥ Conservation & Science, Rijksmuseum, P.O. Box 74888, Amsterdam 1070 DN, The Netherlands

## Abstract

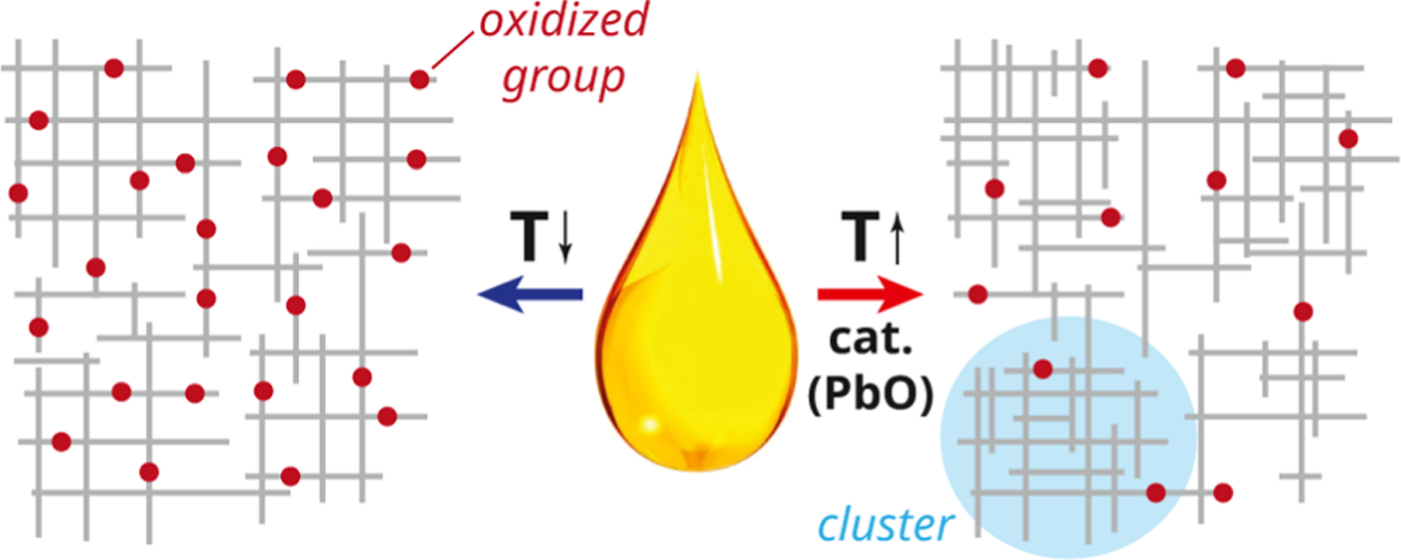

Drying oils like linseed oil are composed of multifunctional triglyceride molecules that can cure through three-dimensional free-radical polymerization into complex polymer networks. In the context of oil paint conservation, it is important to understand how factors like paint composition and curing conditions affect the chemistry and network structure of the oil polymer network and subsequently the links between the structure and long-term paint stability. Here, we employed time-resolved ATR-FTIR spectroscopy and comprehensive data analysis to study the curing behavior of five types of drying oil and the effects of curing temperature as well as the presence of a curing catalyst (PbO). Extracted concentration curves of key reactive functional groups point to a phase transition similar to a gel point that is especially pronounced in the presence of PbO, after which curing reactivity slows down dramatically. Analysis of kinetic parameters suggests that PbO induces a network structure with a more heterogeneous cross-link density, and the ATR-FTIR spectra indicate lower levels of oxidation in those cases. Finally, lower temperatures appear to favor the formation of carboxylic acid groups in oil mixtures with PbO.

## Introduction

Lipids or oils composed of triglycerides can react with atmospheric oxygen in a complex process called autoxidation. While this process is usually undesirable in fat-rich food products or cosmetics, autoxidative polymerization of polyunsaturated vegetable oils (also termed “drying oils”) in oil and alkyd paint formulations is essential to hold pigments together and form a durable paint film. Oil polymerization or curing follows a large number of intersecting reaction pathways which can be affected by environmental conditions like temperature and humidity, as well as paint constituents like pigments, autoxidation catalysts (known as driers), and additives.^1−6^ There are currently still many unresolved questions about the curing kinetics, network structure, and chemical functionality of drying oil polymers. A more complete understanding of oil curing would include not just a comprehensive overview of the many potential reaction pathways but also the evolution of the rates of oxidation and cross-linking reactions as the material transitions from a liquid oil to a polymer network.

There are interesting parallels between drying oil polymers and other polymers which are often formed by uncontrolled free-radical polymerization (FRP) of multifunctional monomers such as polyacrylates and polyacrylamides. In these polymers, it has been observed that cross-link density inhomogeneity arises naturally as a consequence of cyclization reactions (i.e., intramolecular cross-linking in multivinyl monomers or oligomers),^7−11^ which are particularly likely for high-functional monomers such as those in drying oil triglycerides. The kinetics of the polymerization reactions are likely to affect the network topology after the curing phase.^12^ Previous research has discussed a hypothesis for FRP with high-functional monomers where clusters of growing oligomers with high internal cross-link density are formed, which only later link together to percolate and form the polymer network.^13−19^ This cluster formation would be driven by the higher likelihood of forming intramolecular cross-links in oligomers and attachment of fast-diffusing monomers to such clusters, rather than reacting together two slow-diffusing oligomers. Within this model, the level of heterogeneity in the final network is likely to depend on the relative rates of diffusion and cross-link formation. Therefore, it is reasonable to expect that factors that affect the rate of only one of these processes, like the presence of curing catalysts, will have an effect on final network heterogeneity. Three important differences between most previous studies of FRP of multifunctional monomers and curing of drying oils in oil paint are that oil monomers are rather large (approximately 57 carbons or 867 g/mol on average), they are highly functional (typically 5–9 C=C bonds), and they often polymerize in the absence of a solvent.

In this study, the focus is on the link between the overall curing kinetics and resulting polymer network structure, which can provide clues about the dominant reaction pathways under different conditions or in different stages of curing. Specifically, we use attenuated total reflection Fourier-transform infrared (ATR-FTIR) spectroscopy to comprehensively monitor the evolution of functional groups in curing oils, while varying factors that influence curing kinetics (fatty acid distribution, temperature, and the presence of driers).

One of the most common drying oils used in oil paints, linseed oil (LO), has been the focus of previous research on the effects of drying conditions, pigmentation, and the presence of driers on oil polymer formation.^5,20−25^ LO oxidation has also been studied in food science where researchers wish to control the oxidative instability of LO, which impacts its usefulness as a health supplement.^26−28^ Other recent work has investigated the use of LO as a component of self-healing coatings for steels.^29^ Drying oils are comprised of triglyceride molecules with a varying distribution of saturated and unsaturated fatty acid chains, with LO being particularly rich in linolenic acid (C18:3). However, other drying oils like safflower oil (SaO) and poppy seed oil (PO) which are rich in linoleic chains (C18:2) are known to be less prone to yellowing than LO paints. Modern oil or alkyd paint formulations may therefore contain drying oil mixtures or completely replace LO to optimize paint performance.

### Reaction Pathways for Drying Oil Polymerization

**Figure 1:**
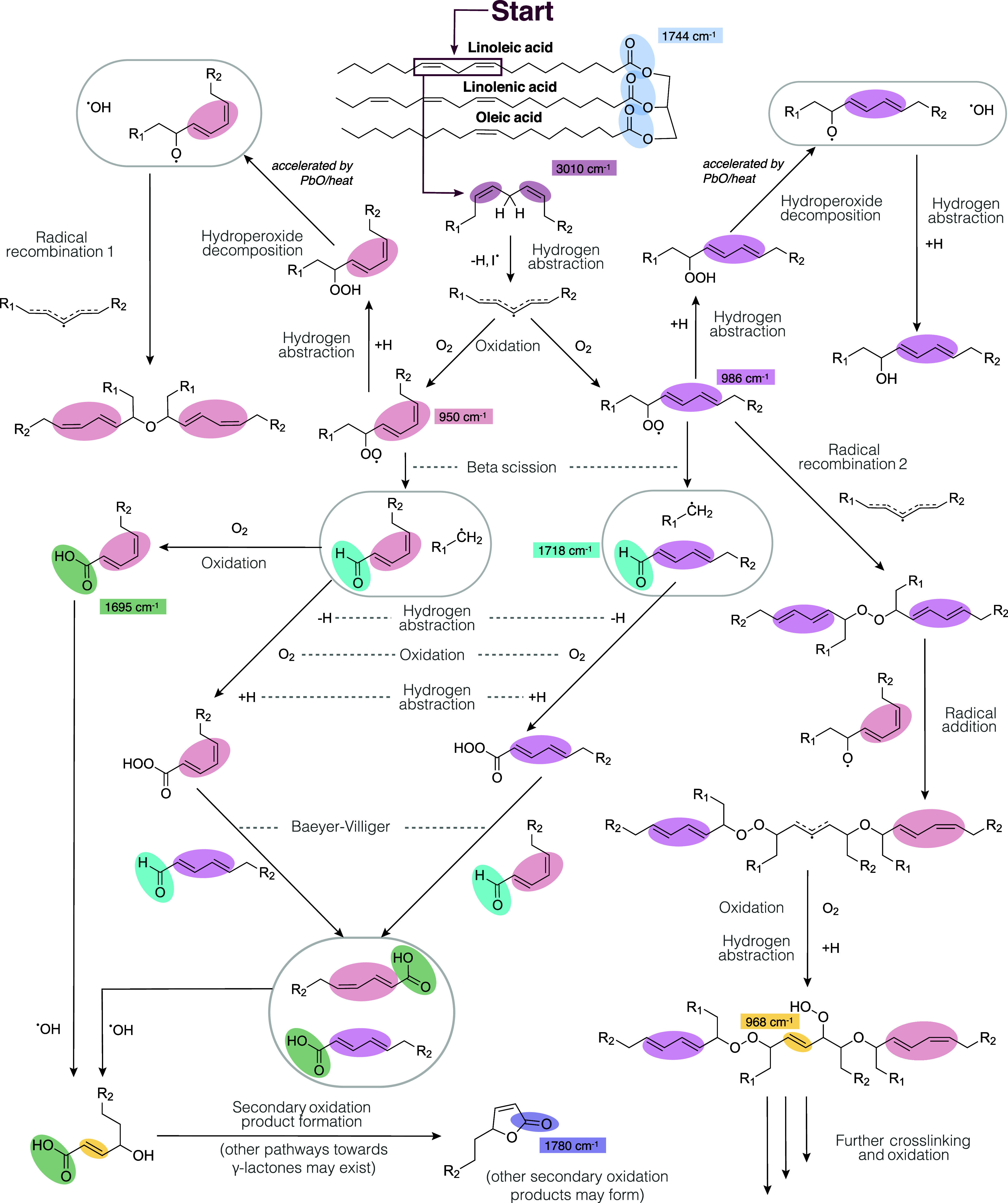
Overview of some of the main reaction pathways during autoxidation of drying oils, taking a linoleic chain as an example. Reactivity starts with abstraction of a bis-allylic hydrogen, after which radical reactions lead to cross-linking, oxygen incorporation, and chain scission. Typical vibrational frequencies of functional groups that can be used as markers for following the curing process are indicated, and these functional groups are color-coded.

To interpret the results of the experiments we report in this work, it is helpful to discuss the general characteristics of drying oil polymerization. Drying oil autoxidation consumes oxygen as part of the propagation step for polymer network formation.^1^ It is virtually impossible to compose a complete overview of the chemical reactions that cause curing of drying oils, even with the help of computational methods to map out the reaction network.^6,30^ A partial reaction scheme is shown in Figure 1 that highlights examples of some dominant chemical reactions and vibrational frequencies of functional groups relevant for the experimental work we report.

At the start of the curing process, a radical initiator abstracts a weakly bonded hydrogen atom between a set of nonconjugated *cis* C=C bonds in the fatty acid chains (a bis-allylic hydrogen). The resulting carbon radical can react with molecular oxygen to form a peroxide radical (−OO^·^) and a set of conjugated C=C bonds. Here, the peroxide radicals can undergo several reactions, leading to a split in the reaction pathways. Possible reactions include forming a hydroperoxide via hydrogen abstraction, radical recombination reactions, or beta-scission reactions to form a new carbon radical and an aldehyde. The newly formed hydroperoxide can be decomposed to form an alkoxy radical (−O^·^) and a hydroxide radical (OH^·^), which is thought to be especially effective as a radical initiator due to its small size. The rate of hydroperoxide decomposition can be accelerated by either elevated temperatures or the addition of metal-based driers. These driers typically strongly decrease the induction time that is caused by the presence of antioxidants naturally present in drying oils, as well as accelerating the overall curing kinetics.^4,5^ Since many of the formed carbon radicals are situated near the unsaturations on the fatty acid chains, these C=C bonds easily undergo isomerization reactions, leading to populations of *cis*–*trans* and *trans*–*trans* conjugated C=C bonds. All recombination and addition reactions are possible with both types of conjugated C=C bonds, but for clarity, not all possibilities are shown. Through either radical recombination or radical addition, a proportion of carbon or oxygen radical species reacts to form oligomers that, over time, combine to form a polymer network. Three types of cross-links are present in the final polymer network: peroxy (C–O–O–C), ether (C–O–C), and alkyl (C–C, not shown in Figure 1) cross-links.^4^

In the curing phase, the reacting oil is very active and can undergo many chemical conversions alongside the C=C bond reactions. For example, primary and secondary alcohols formed after radical initiation and oxidation can be oxidized to form aldehydes, ketones, carboxylic acids, and eventually secondary oxidation products. The formation of these secondary oxidation products has been inferred by the observation of a band in the FTIR spectra of drying oils at around 1780 cm^–1^.^5^ In Figure 1, we show the formation of a γ-lactone as an example of a secondary oxidation product, formed in a ring-closing reaction of an alcohol-substituted carboxylic acid. The unsaturation present in the γ-lactone ring can shift the carbonyl stretch vibration band to between 1773 and 1780 cm^–1^.^31^ In theory, γ-lactones could be formed by other (radical-mediated) reaction pathways as well. Several other secondary oxidation products with carbonyl vibrations around 1780 cm^–1^ have also been proposed.^20,23,32^ We included the γ-lactone as a representative product in this scheme because the existence of alcohol-substituted carboxylic acids is likely in the curing phase of oils and because the ring-closing reaction to the lactone would be thermodynamically favorable. As the oxidation reactions progress, the polymer network becomes increasingly polar, which is believed to make drying oil polymers more susceptible to damage during solvent or water exposure.^33,34^

While autoxidation reactions such as those illustrated in Figure 1 will slow down as the concentrations of reactive species diminish and the system becomes increasingly cross-linked, they do not fully stop. In the curing phase of oil reactivity, which is the focus of the current work, network formation reactions dominate over scission reactions and ester hydrolysis. However, on the long term, in what we will call the aging phase, those latter processes will start to dominate and the cross-link density may decrease, often leading to detrimental changes in material properties. If cured oil paints can have a variable degree of heterogeneity in cross-link density, one may also expect differences in the rate of material deterioration as a consequence of network breakdown reactions.

While there have been claims in the literature that the fatty acid distribution of drying oils does not impact the polymer network structure at the end of the curing phase,^21^ we suspect that the extent and spatial distribution of cross-links and oxidized functional groups within the oil network depends on initial fatty acid distribution (i.e., monomer functionality). In support of this idea, a recent study by Švarcová et al. investigated mixtures of minium (Pb_3_O_4_) and four different drying oils with X-ray diffraction.^35^ They showed that the oils exhibited strong differences in the concentration and size of polymer domains rich in amorphous lead carboxylates, which are a product of the reaction between minium and the oils. This variation in the behavior of amorphous lead carboxylates in the oil network implies that either the formation kinetics and/or the network properties that influence lead carboxylate organization are directly affected by the distribution of fatty acids participating in oil polymerization.

### Research Approach

Many analytical techniques have been used to monitor the chemical evolution of drying oils in the curing phase, including FTIR spectroscopy, nuclear magnetic resonance (NMR) spectroscopy, differential scanning calorimetry (DSC), size exclusion chromatography (SEC), gas chromatography paired with mass spectrometry (GC–MS), and thermogravimetric analysis (TGA).^5,20−26,34,36−45^ NMR spectroscopy has been utilized to determine the initial composition of drying oils,^42^ but full characterization of the polymerization process with NMR spectroscopy becomes challenging as the oil solidifies with an increasingly diverse functional group population. Nevertheless, solid-state NMR spectroscopy has been used successfully to measure spin relaxation times in a cured oil polymer network as a measure for the extent of cross-linking.^34^ DSC has been useful to study the effect of antioxidants on the induction time of drying oils as well as to estimate concentrations of radical-forming peroxides in the oil network.^26,43^ However, in complex systems like drying oils, DSC requires a second technique to identify the nature of observed thermal processes, and being limited to thermal effects, it has a rather narrow scope. Similarly, TGA can be used to quantify the rate of oxygen consumption and the evaporation of volatiles, but the chemical functionality of the evolving polymer network remains hidden.^43,45^ SEC and GC–MS are typically applied to characterize early stage oligomers or the solvent-extractable components of the cured oil network,^20,37,40,46^ or the polymer network can be investigated by GC–MS after fragmentation by pyrolysis (py-GC/MS).^25,34,43,44^ In each of these cases, however, one obtains information on only a subset of the curing oil that may not be representative of the whole system, making it rather challenging to relate measured fragment distributions to the network structure in a quantitative way. While FTIR spectroscopy has limited sensitivity, it is very suitable to monitor the evolving composition of a curing oil with high levels of chemical specificity throughout the entire transformation from a liquid oil to a polymer network. Several researchers have charted the vibrational bands that change in intensity and position in the curing phase. Most band assignments in the current study work were confirmed via these reports.^5,20−23,36,38,39,41,42^

Here, we expand on previous work by using ATR-FTIR spectroscopy at high time resolution and long curing times to monitor the FRP process in five types of drying oils (linseed, safflower, walnut, poppy seed, and stand). Stand oil (StO) is a processed LO that is oligomerized by subjecting the oil to high temperatures (∼300 °C) in an anoxic environment.^47^ By using a higher time resolution than most previous reports, as well as rigorous spectral analysis using difference spectra, band integration, and deconvolution, detailed analyses of polymerization kinetics become possible which reveals aspects of the polymer network structure. We focused on the addition of a drier (lead oxide, PbO) and temperature as ways to modify the kinetics of curing.

## Experimental Section

### Materials

Five drying oils were used in this study: cold-pressed LO (Kremer Pigmente), cold-pressed extra pale walnut oil (WO, Chelsea Classical Studio), StO (Van der Linde Kunstenaarsmaterialen), SaO (Verfmolen “De Kat”), and purified PO (Talens). Lead(II) oxide (PbO) was purchased from Alfa Aesar, which was predominantly β-PbO (massicot) mixed with smaller amounts of α-PbO (litharge), as determined by X-ray diffraction.

### Methods

The fatty acid composition of the drying oils was calculated using ^1^H and ^13^C NMR spectra. Acid values were determined using a titration protocol adapted from AOCS Official Method Ca 5a-40. Further details for both these methods are described in Supporting Information Section A. ATR-FTIR spectroscopy was performed using a PerkinElmer Frontier spectrometer equipped with a heatable diamond GladiATR module (Pike Technologies). After preheating the ATR module to the desired temperature for a particular run, a small droplet of oil was applied on the ATR diamond to create a thin film, ensuring full coverage. Spectrum collection was started immediately, from 450 to 4000 cm^–1^ using a single scan and 4 cm^–1^ resolution under isothermal conditions. The spectra were measured every 1–5 min for up to 20–90 h, or until the complete disappearance of the band at 3010 cm^–1^ (*cis* C=C–H stretch), which can be seen as the end of the initial curing phase. For samples containing PbO, 5% (w/w) PbO was added to the oil, after which the mixture was ground in a mortar and pestle for 5 min until it was smooth.

## Results and Discussion

### Characterization of Oil Composition

The acid values and fatty acid distributions (see Supporting Information Section A) in the five drying oils are presented in Table 1. The five drying oils can be split into three categories: oils containing linolenic acid (LO and WO), oils containing mostly linoleic acid (SaO and PO), and prereacted oil (StO). The prereaction of StO leads to the formation of oligomers, which means that the initial fatty acid distribution can no longer be determined. The acid values provide information about the initial concentration of free fatty acids in the oils, which may influence oil reactivity. For LO and WO, the acid values (corresponding to ∼3 mol % of free fatty acids) are fairly similar, which may be due to the fact that both oils were cold-pressed. The acid value for PO is lower than LO and WO, most likely due to the purification process specified by the supplier.

**Table 1:** Acid Values and Fatty Acid Distributions of LO, WO, PO, SaO, and StO^a^

drying oil	acid value (mg_NaOH_/g_oil_)	C18:3 (%)	C18:2 (%)	C18:1 (%)	C18:0/C16:0 (%)	[cis C=C] NMR^b^	[cis C=C] ATR-FTIR^b^
LO	1.67 ± 0.02	53	19	18	9.8	1.0	1.0
WO	1.54 ± 0.07	16	55	19	10	0.82	0.83
PO	0.40 ± 0.01	0	75	15	10	0.77	0.78
SaO	1.10 ± 0.03	0	75	15	9.6	0.77	0.75
StO^c^	4.19 ± 0.06	—	—	—	—	—	0.29

a The acid values are reported with the standard deviation of triplicate measurements.

b Concentrations of *cis* C=C bonds are normalized with respect to their concentration in LO.

c The fatty acid distribution of StO cannot be determined due to its prepolymerization.

The fatty acid distribution in the oils may have important consequences for the kinetics of the curing phase of the oil. The oils with high linolenic acid concentration (especially LO) are expected to show more rapid initial curing kinetics because their higher concentration of bis-allylic hydrogens provides many radical initiation sites. The prepolymerization of StO means that it probably cures much slower than the other oils due to a low concentration of bis-allylic hydrogens and its high initial viscosity.

**Figure 2:**
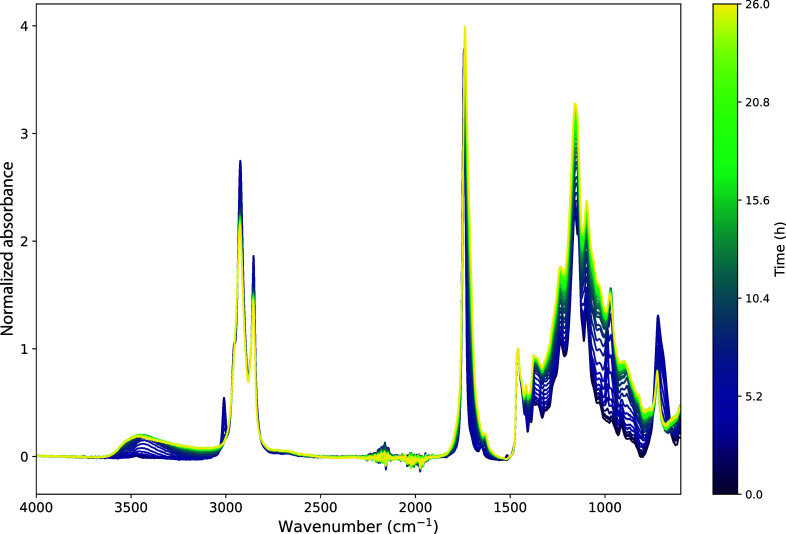
ATR-FTIR spectra collected during curing of WO at 70 °C. The spectra were baseline-corrected using a set of linear baselines connecting the spectral values at 3900, 2500, 1900, and 1500 cm^–1^ and were normalized to the δ CH_2_ band at 1465 cm^–1^.

Figure 2 provides an example of a selection of ATR-FTIR spectra collected for WO heated at 70 °C, showing that there are either chemical conversions or concentration changes in nearly all functional groups in the oil. The initial and final spectra after curing for all oils are shown in Figure S11. Data sets like these were used to carefully monitor intensity changes of specific features and make comparisons between the oils and between curing conditions, providing clues about curing kinetics, polymer network structure, and oil oxidation.

### Kinetics of *cis* C=C Consumption

**Figure 3:**
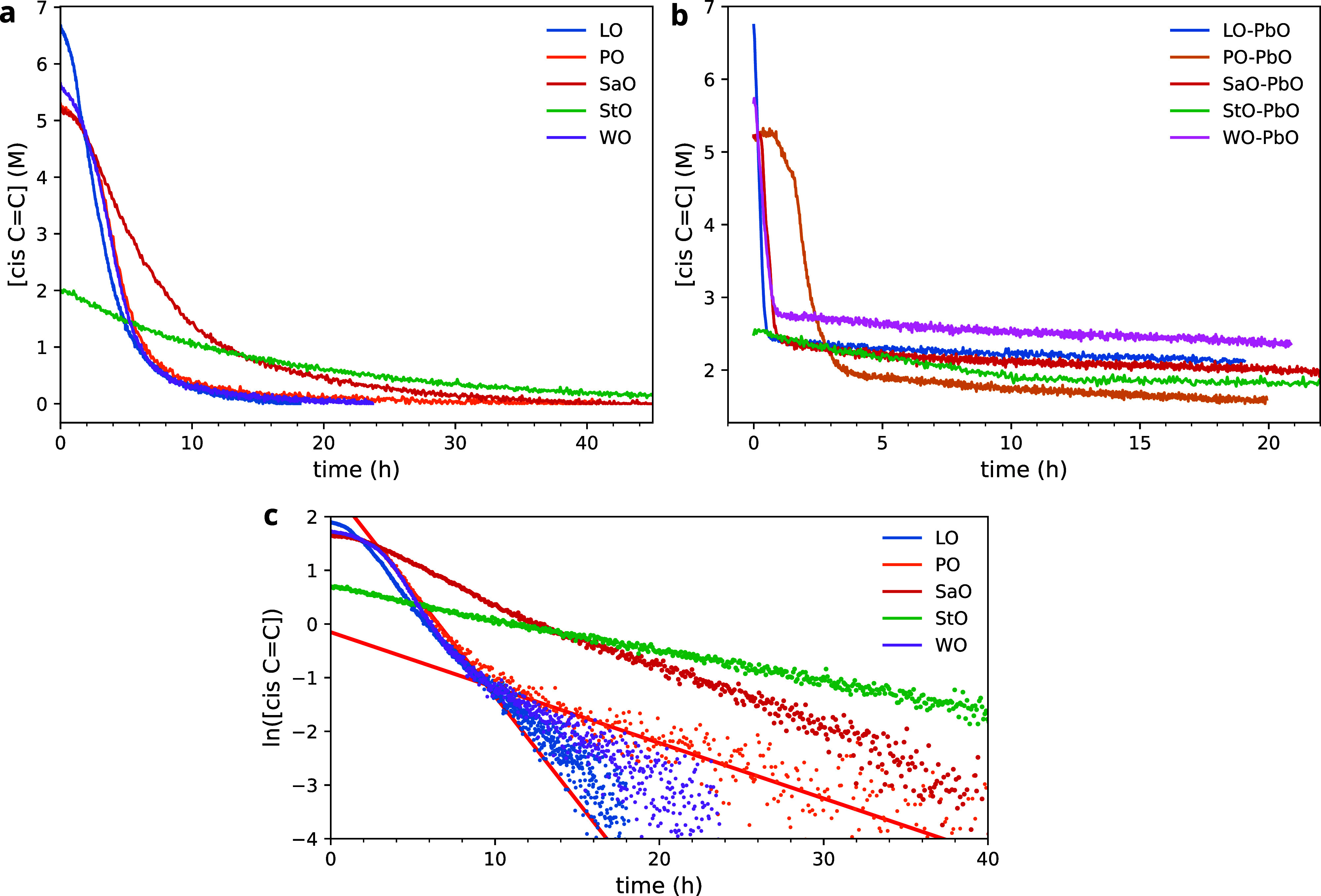
Comparison of the concentration of *cis* C=C bonds over time at 70 °C for a (a) set of drying oils and the (b) same oils with 5% (w/w) PbO as a drier, as calculated from the ν(C=C–H) band at 3010 cm^–1^. (c) Same data as in (a) plotted as the natural logarithm of [*cis* C=C]. Red lines indicate the best linear fit to the two linear domains of data corresponding to PO. In (a,c), the profiles were shifted to remove the induction time, i.e., *t* = 0 at the start of *cis* C=C consumption.

A key feature in the FTIR spectra of curing oils, such as those in Figure 2, is the rapid reduction of the band at 3010 cm^–1^ associated with the C–H stretch vibration in *cis* C=C bonds. This band is reduced as a consequence of C=C bond isomerization and conjugation after bis-allylic hydrogen abstraction (see Figure 1). Figure 3 shows the concentration profiles of *cis* C=C bonds associated with the 3010 cm^–1^ band for various oil and oil/PbO mixtures. All oils exhibited an induction time, observed as a (nearly) horizontal section in the concentration curves at short curing times, which are due to varying concentrations of antioxidants naturally present in the oils. This induction time does not affect the oil polymerization process, so the curves were shifted to set *t* = 0 at the start of *cis* C=C consumption to allow easy comparison of the time scales of curing. Since the initial fatty acid composition is known from NMR spectroscopy (Table 1), and all profiles continued to complete conversion of *cis* C=C bonds, vibration band intensities could be converted to real concentrations of *cis* C=C bonds. The associated relative initial concentrations of C=C bonds in this set of oils are in very good agreement with the results from NMR calculations (Table 1). The calculation of *cis* C=C concentrations from ATR-FTIR data relies on assuming a value for the density of the oils and assuming that this density does not change throughout the curing process. While these assumptions are not strictly true, they allow very useful comparison of the reaction rate estimates.

To investigate the kinetic behavior of *cis* C=C consumption, Figure 3c shows the profiles of ln([*cis* C=C]) for the five oils. While StO shows a nearly perfect linear trend, the other oils all exhibit two linear domains with a different slope. The existence of such clear linear domains indicates that *cis* C=C consumption is well-described as a first-order kinetic process. Rate parameters were estimated by linear fits to the two linear domains, extracting a fast and slow rate constant, *k*_fast_ and *k*_slow_. The transition point between the two domains was defined as the conversion of *cis* C=C at the intersection of the fitted lines, calculated as
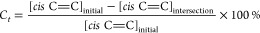


All relevant kinetic parameters are listed in Table 2, and fits for all samples are shown in Figures S8–S10. In these drying oils, *k*_fast_ and *C*_t_ were very similar for LO, WO, and PO, despite their differences in fatty acid composition. There was greater variation in *k*_slow_, although the rate constant decreased with a factor of 4 at most for PO.

**Table 2:** Kinetic Parameters for *cis* C=C Consumption during Oil Curing

sample	T (°C)	k_fast_ (h^–1^)	k_slow_ (h^–1^)	C_t_(%)
LO	70	0.40	0.28	89
WO	70	0.46	0.18	92
PO	70	0.39	0.10	91
SaO	70	0.15	0.11	80
StO	70	0.061	0.058	39
LO–PbO	70	2.2	0.0056	65
WO–PbO	70	0.97	0.0072	52
PO–PbO	70	0.57	0.013	63
SaO–PbO	70	1.2	0.0066	57
StO–PbO	70	0.028	0.0022	24
LO–PbO	30	0.12	0.022	57
LO–PbO	40	0.17	0.022	57
LO–PbO	50	0.48	0.0068	76
LO–PbO	60	0.43	0.011	65
LO–PbO	70	2.2	0.0062	65

Carrying out the same analysis for the oil mixed with 5 wt % PbO (Figure 3b and Table 2), it is immediately clear that the addition of PbO has very interesting effects on the oil curing process. PbO accelerated the fast domain of *cis* C=C consumption by a factor of more than 5 for LO, but this effect was not equally strong for all oils. In fact, PbO had only minor effects on the fast curing kinetics in PO, and it seems to have slowed down the curing of StO. The most striking effect, however, is that PbO drastically reduced the value of *k*_slow_ and moved the transition point to much lower conversions, in the range of 52–65% rather than values close to 90%.

The change in reaction rates at *C*_t_ points to a rather sudden change in the structural properties of the growing polymer network, a phase transition similar to the concept of a gel point. After the gel point, the sudden increase in viscosity will decrease the diffusion rate of radical initiators and reaction products, leading to overall slower *cis* C=C consumption. Comparing the curing behavior of the oils with and without PbO, it is curious that fast initial reactivity is anticorrelated to second-stage kinetics. For example, even though LO reaches the gel point already at 65% conversion with PbO compared to 89% without PbO and initial curing is a factor of 5 faster with PbO, the corresponding *k*_slow_ is a factor of 50 slower with PbO. This observation suggests differences in the polymer network structure caused by PbO that affect the diffusion rates. Somewhat paradoxically, the oils with PbO seem to have an overall lower cross-link density (evidenced by considerably lower *C*_t_), while also having slower overall reaction kinetics after the gel point (evidenced by a much lower *k*_slow_). These observations may be explained by concluding that the cross-link density is far more heterogeneous in oils that contain PbO. The existence of clusters with very high cross-link density or high concentrations of intramolecular cross-links, and associated very slow diffusion, would drastically reduce the reaction rate of unreacted *cis* C=C bonds. Previous researchers have already suggested a link between fast polymerization kinetics and the formation of a more clustered polymer network.^15^ As a consequence of this clustering, there is considerable latent reactivity still present in oils cured with PbO, in the order of 2–3 M *cis* C=C after 20 h of curing at 70 °C. It is also interesting to note that the presence of two distinct reaction rate coefficients for curing of LO, WO, and PO suggests that, even when starting with homogeneous mixtures, a polymer network with some level of heterogeneity may form spontaneously.

**Figure 4:**
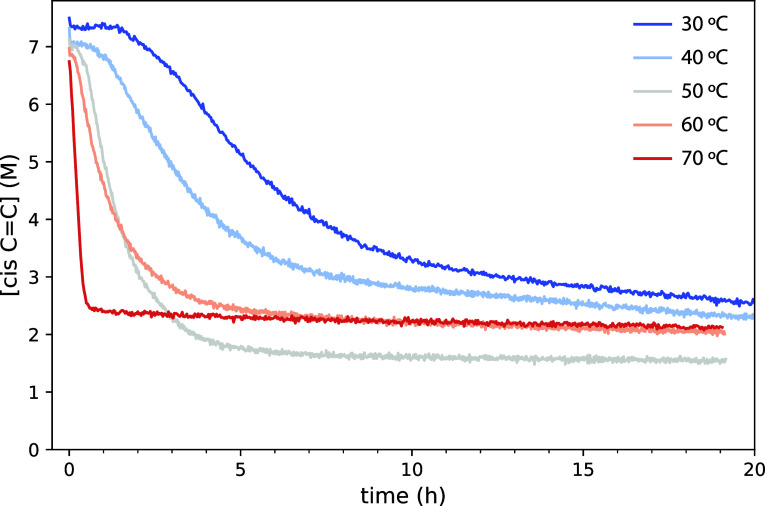
Concentration profiles for *cis* C=C bonds in LO with 5 wt % PbO time at 30–70 °C, as calculated from the ν(C=C–H) band at 3010 cm^–1^.

To investigate the link between the curing kinetics and network structure further, it is interesting to consider the *cis* C=C concentration profiles of LO–PbO mixtures as a function of temperature (Figure 4). It is evident that lower temperatures lead to slower curing kinetics. However, applying the same two-domain kinetic fit as above (Figure S10), it appears that only the first curing stage governed by *k*_fast_ has clear sensitivity to temperature (Table 2). The second slower stage after the gel point is not so strongly affected by temperature. If anything, the second stage is faster at lower temperatures. A similar temperature series for PO–PbO mixtures showed the same effect (not shown). This rather curious result demonstrates the complexity of oil curing, especially when catalysts are involved. The weak temperature dependence of *k*_slow_ also supports the notion that the kinetics of curing in the slow stage are largely diffusion-controlled. The temperature dependence of *k*_fast_ allows us to estimate an apparent activation energy of *cis* C=C consumption in LO–PbO at 57 ± 13 kJ/mol. LO–PbO curing at 30 °C is approaching a more realistic scenario of oil curing in oil paintings, which usually occurs at ambient temperature and involved the use of driers. Under such conditions, Figure 4 indicates that one may expect a rather early gel point (*C*_t_ in the range of 55–60%), leading to a high concentration of reactive bis-allylic hydrogens after the gel point, a network with considerable cross-link heterogeneity, and a long reaction time on the order of 8–12 days to complete *cis* C=C conversion despite the presence of a catalyst.

### C=C Bond Isomerization

Once the FRP reaction in drying oils is initiated by bis-allylic hydrogen abstraction, the initial population of almost exclusively nonconjugated *cis* C=C bonds is slowly consumed and isomerized, with the formation of conjugated *cis–trans* and *trans–trans* C=C bonds as a consequence (see Figure 1). After further radical addition and oxidation reactions, nonconjugated *trans* C=C bonds form which, due to their limited reactivity, may persist for a much longer time. The spectral signatures for these C=C bond variations occur between 930 and 1000 cm^–1^ in the region for the C=C–H wagging vibrations. There are three bands of interest within this region: the band at 986 cm^–1^ attributed to conjugated ω(*trans–trans* C=C–H), the band at 968 cm^–1^ attributed to nonconjugated ω(*trans* C=C–H), and the band at 950 cm^–1^ attributed to conjugated ω(*cis–trans* C=C–H).^5,36^ These overlapping three bands were modeled with a linear combination of Gaussian band shapes (see Supporting Information Section B), allowing monitoring of individual C=C species over time using the area of the modeled bands.

**Figure 5:**
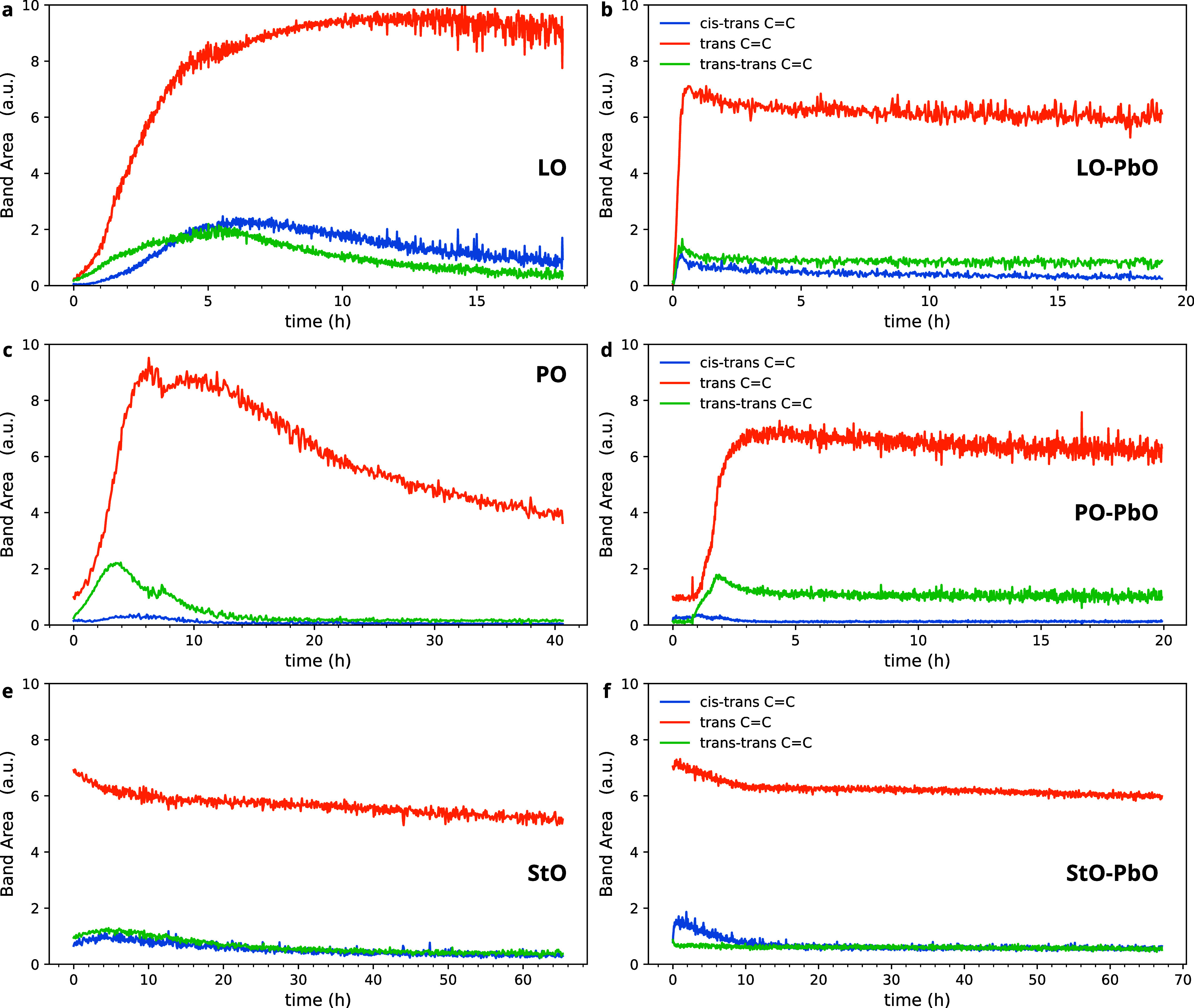
Comparison of the band areas corresponding to ω(CH) bands at 950 cm^–1^ (*cis–trans* C=C), 968 cm^–1^ (*trans* C=C), and 986 cm^–1^ (*trans–trans* C=C). The band areas are shown for (a) LO, (b) LO–PbO, (c) PO, (d) PO–PbO, (e) StO, and (f) StO–PbO, all measured at 70 °C. A similar figure containing data for WO and SaO can be found in Figure S12 in the Supporting Information.

The three ω(C=C–H) bands provide important insights into the autoxidation pathways beyond the stage of initiation and *cis* C=C consumption. Figure 5 provides a comparison of the band areas for the ω(C=C–H) for LO, PO, and StO, both with and without PbO. For LO, the bands associated with newly formed C=C groups appeared almost immediately after the decrease in nonconjugated *cis* C=C concentration (compare Figures 3a and 5a). At this point, the concentration of nonconjugated *trans* C=C bonds grew rapidly, while the conjugated C=C bond concentrations went through a weaker maximum around 5 h after the start of curing, after which the concentration decreased to nearly zero. The immediate formation of isolated *trans* C=C bonds and only low concentrations of conjugated C=C bond pairs indicates that the reactions illustrated in Figure 1 occur very rapidly once the autoxidation process is initiated by H abstraction. The isolated *trans* C=C bond appears to be much less reactive and persists on a longer time scale.

PO showed similar behavior with a strong *trans* C=C bond concentration (Figure 5c). In contrast to LO, there was almost no *cis–trans* C=C formed in PO. Similar observations were made for WO and SaO (see Figure S12a,c). Since PO, WO, and SaO contain little to no linolenic acid chains (see Table 1), this difference suggests that linolenic acid with its three C=C bonds is more likely to isomerize to *cis–trans* C=C bonds than linoleic acid. The concentration of *trans* C=C bonds decreased by approximately half over the course of 30 h after a maximum was reached, again similar to WO and SaO. This decrease indicates that, while slower to react than conjugated C=C bonds, isolated *trans* C=C will be consumed by autoxidation processes eventually. Being prepolymerized, StO started out with a dominant *trans* C=C bond concentration and only very weak conjugated C=C contributions (Figure 5e). Though the conjugated bonds reached a small maximum due to additional consumption of initial *cis* C=C bonds, somewhat unexpectedly, the isolated *trans* C=C bond only decreased.

The addition of PbO had a strong effect on the evolution of C=C species in drying oils (Figure 5b,d,f), similar to what was observed in Figure 3b. Immediately when the initial *cis* C=C bonds start to be consumed, there was a corresponding rapid increase in especially the isolated *trans* C=C bond concentration in LO–PbO and PO–PbO mixtures. Similar to Figure 3b, there was a sharp transition early in the autoxidation process, after which there was only minor decrease in all C=C bond concentrations. WO and SaO showed a similarly sharp transition as LO when mixed with PbO (Figure S12b,d), while StO reactivity appeared similar with or without PbO (Figure 5f). These observations support the idea that there is a clear phase transition in the oils when they are cured at elevated temperatures with PbO, after which both autoxidation initiation and C=C conversion reactions become exceptionally slow. The long persistence of potentially reactive C=C species under these conditions suggests that local cross-linking prevents diffusion of reactants and a network architecture that is different from oil cured without a catalyst. Due to their relative stability, it is expected that the *trans* C=C bonds only react significantly with radical species.^37,48^ These species can be alkoxy radicals (leading to cross-link formation) when conditions allow it. However, when PbO is present and the polymer becomes more densely cross-linked, it may be that only small radical species like ^·^OH contribute significantly to *trans* C=C bond conversion, as alkoxy radicals tend to be far larger molecules characterized by slow diffusion.

As expected from the analysis of *cis* C=C consumption, lower temperatures depress the rate of C=C isomerization reactions in LO–PbO (Figure S13). More surprising is that a higher concentration of *cis–trans* C=C and *trans–trans* C=C bonds is built up at lower temperatures. This different concentration ratio of C=C types at intermediate curing times implies that not all reactions in the autoxidation pathway are equally dependent on temperature and that the balance of cross-link types and other newly formed functional groups may be sensitive to temperature.

Detailed tracking of the reaction products of *trans* C=C consumption using ATR-FTIR spectroscopy is currently not feasible, because at this point the reaction pathways become too diverse and the resulting species have almost no unique vibrational features. However, it is interesting to consider in general the vibrational features related to C=O and C–O bonds to monitor the formation of oxidation products.

### Oxidation Products Formed during Curing of Drying Oils

**Figure 6:**
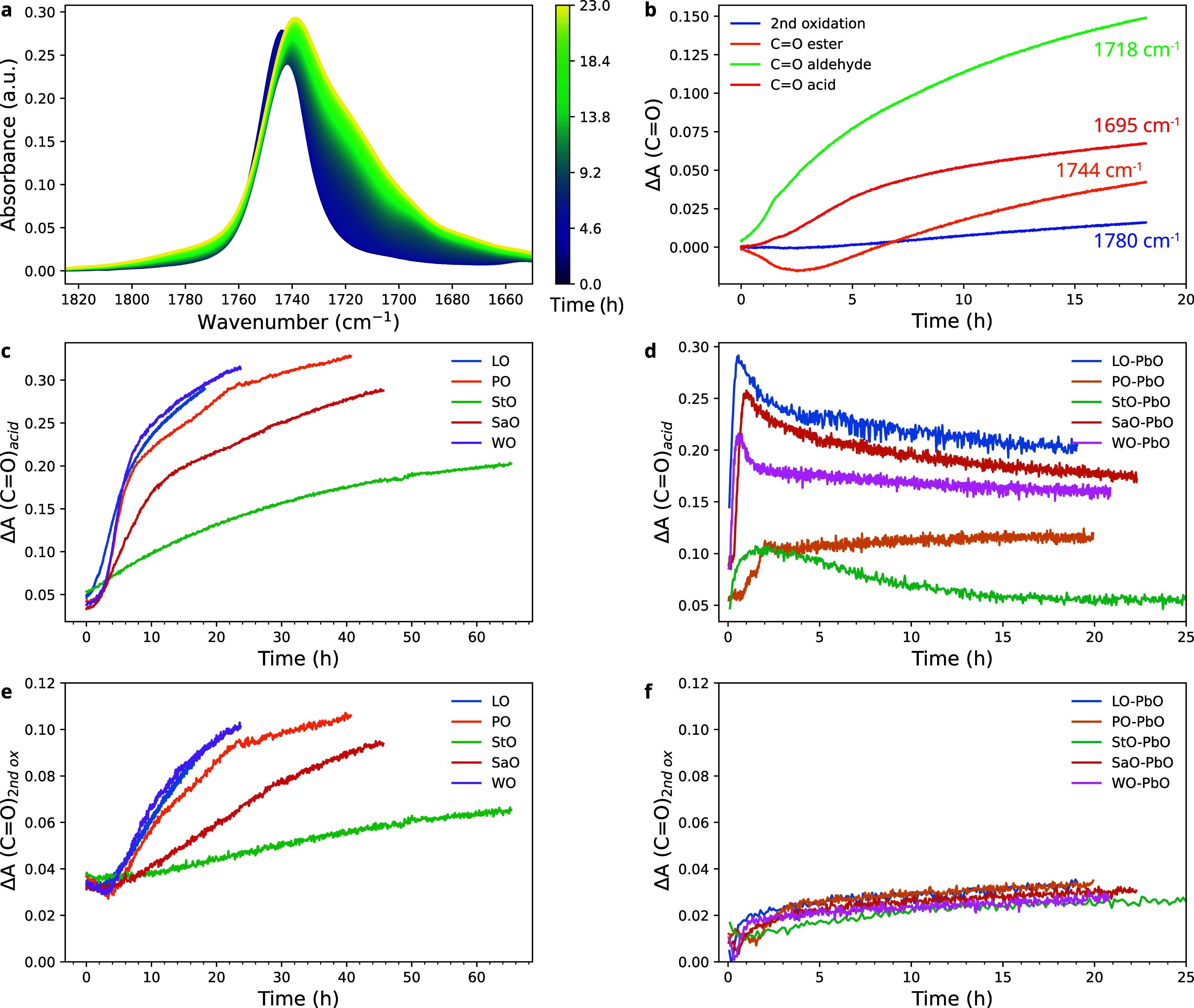
(a) Evolution of the carbonyl region over time for LO at 70 °C. (b) Change in absorbance for each of the ν(C=O) band contributions for LO shown in (a). The data (b) represents the differences from the initial absorbance at each band center frequency. Comparisons of the ν(C=O)_acid_ at 1695 cm^–1^ for the (c) oils and (d) oil mixtures with PbO and the ν(C=O)_second ox_ at ∼1780 cm^–1^ for (e) oils and (f) oil mixtures with PbO. Profiles shown in (c–f) were normalized to the initial intensity of the ν(C=O)_ester_ at 1744 cm^–1^. Data shown (a–c,e) were baseline-corrected at 1850 cm^–1^, while (d,f) were baseline-corrected using a line drawn between 1850 and 1810 cm^–1^. In (b,c,e), the profiles were shifted to remove the induction time, i.e., *t* = 0 at the start of *cis* C=C consumption.

The C=C bonds in fatty acid chains can react with oxygen-containing radicals to form ether and peroxide cross-links, but there are competing pathways that lead to oxygen-containing functional groups like aldehydes, carboxylic acids, and possibly lactones (Figure 1). The carbonyl region in the FTIR spectra (1650–1800 cm^–1^) is a useful starting point to gain some insights into oxidation products in drying oils. Figure 6a shows the evolution of the carbonyl region of LO during curing at 70 °C. The C=O stretch vibration was initially centered at 1744 cm^–1^, corresponding to the ester group in the triglycerides (ν(C=O)_ester_). As the oil cures, bands associated with carbonyl stretches in aldehydes (ν(C=O)_aldehyde_) and carboxylic acids conjugated to a C=C bond (ν(C=O)_acid, conj_) increased at 1718 and 1695 cm^–1^, respectively, as judged by second-derivative spectra (see Figure S14).^5,22^ On a longer time scale, growth of a shoulder centered at 1780 cm^–1^ was observed that is usually associated with “secondary oxidation products” ν(C=O)_second ox_), one of which is hypothesized to be a γ-lactone.^38^ We made an attempt to model these overlapping carbonyl bands with a linear combination of Gaussian band shapes, similar to the approach followed for analyzing C=C isomerization reactions. However, due to the large number of overlapping bands in a narrow frequency region and changes in band positions and widths in the course of curing, meaningful fits required too many degrees of freedom to obtain a stable result (see Section B2 in the Supporting Information for details). Instead, the intensities at four carbonyl band positions are plotted in Figure 6b–f. Figure 6b shows the change in the ν(C=O) bands in LO. In this figure, the shift of ν(C=O)_ester_ from 1744 to 1740 cm^–1^ caused by a decrease in ester concentration and increased aldehyde and acid contribution resulted in a dip in the ester C=O profile. There was a clear sequence in the formation of the different oxidation products, which matches with their increasing number of required reaction steps away from the initial fatty acid chain structure. The ν(C=O)_aldehyde_ band increased simultaneously with *cis* C=C consumption (Figure 3a) and C=C isomerization (Figure 5a), at ∼1 h. The ν(C=O)_acid, conj_ band began to increase about 1 h later, with a slower rate than the ν(C=O)_aldehyde_ band. At ∼ 6 h, the growth of the secondary oxidation product band started but to a much lesser extent than the other ν(C=O) bands.

The oxidation behavior of the various oils and the effects of PbO are compared in Figure 6c–f. Since the overall shape of the profiles for ν(C=O)_aldehyde_ and ν(C=O)_acid, conj_ bands was similar, only the ν(C=O)_acid, conj_ profiles are shown. Comparing the oils, the trends in Figure 6c,e are very similar, although StO formed carboxylic acid groups more slowly and to a lesser degree than the other oils. The band associated with secondary oxidation products grew consistently later and to a lesser degree than the ν(C=O)_acid, conj_ bands, confirming that these products are formed further down the curing pathway. In line with previous observations, the addition of PbO led to lower levels of curing reactivity, in this case resulting in lower concentrations of carbonyl species (Figure 6d,f). The ν(C=O)_acid, conj_ band shows a decrease after a rapid increase at short time scales because the carboxylic acids reacted rapidly with PbO to form lead carboxylates. A corresponding increase in asymmetric lead carboxylate C–O stretch vibration bands was observed between 1500 and 1640 cm^–1^ (Figure S15 and Section E in the Supporting Information). While there were clearly several distinct lead carboxylate species formed in the course of our experiments, the differences between the PbO–oil mixtures with regard to lead carboxylate formation remain rather puzzling, and they will not be discussed further in the context of this paper. The ν(C=O)_second ox_ band growth was reduced under these conditions of lower acid concentration (caused by PbO), which is in line with the hypothesis that γ-lactones are partly responsible for this band. Therefore, despite the accelerated initial curing kinetics in PbO-containing systems, PbO appears to lead to a less-oxidized oil polymer after curing. This observation agrees with findings by Pizzimenti and co-workers, who reported fast curing but low levels of oxidation in paint films containing lead white pigment.^25^

**Figure 7:**
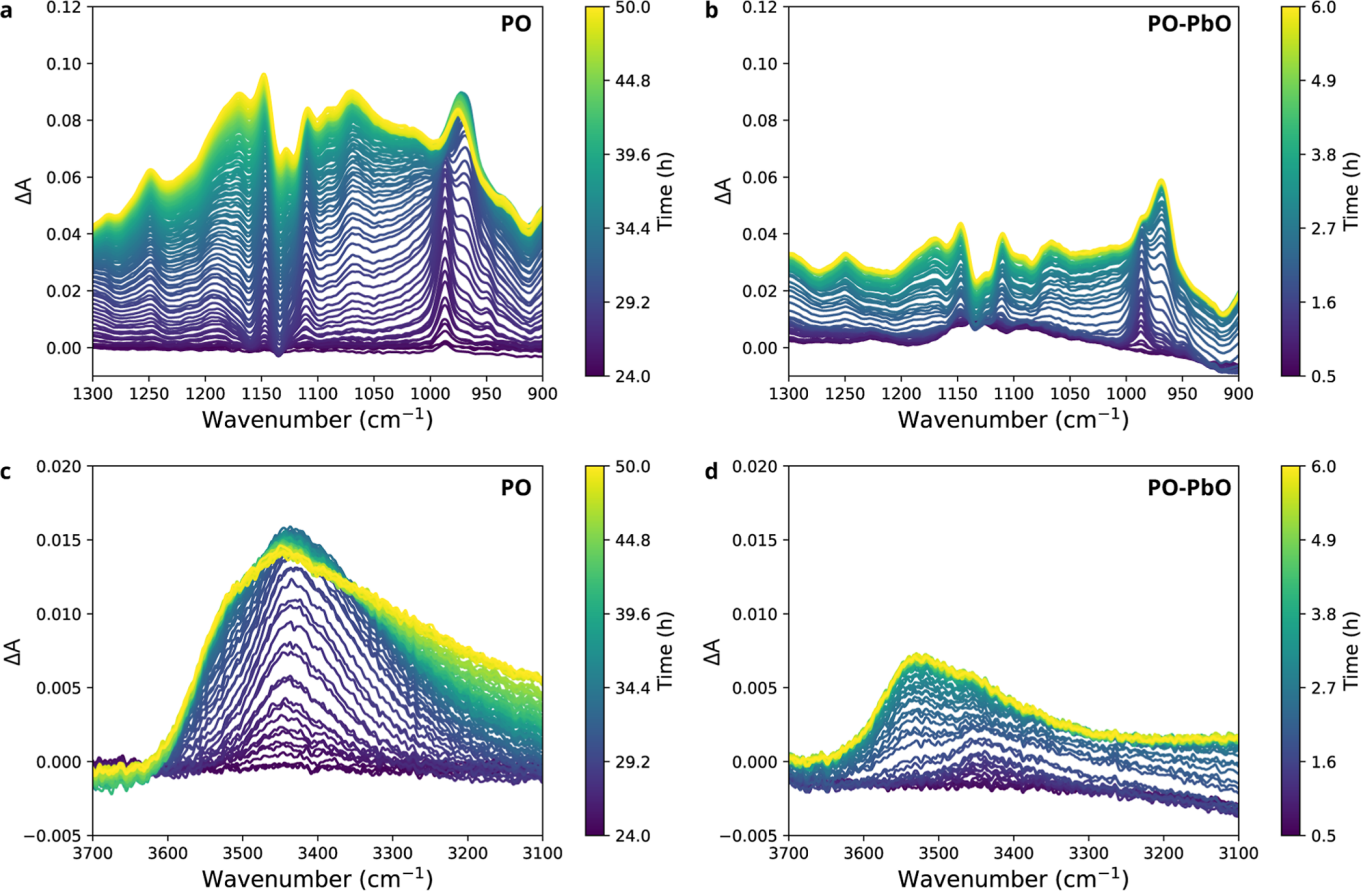
ATR-FTIR difference spectra showing the changes at 70 °C in the C–O stretch vibration regions for (a) PO and (b) PO–PbO and the changes in the O–H stretch vibration region for (c) PO and (d) PO–PbO. The spectra were baseline-corrected prior to the calculation of difference spectra.

A similar conclusion about a lower degree of oxidation under catalyzed curing conditions is reached when considering other regions of the ATR-FTIR spectra. Both the C–O stretch vibration bands in the region 1000–1200 cm^–1^ and O–H bands between 3100 and 3600 cm^–1^ were increasing during curing due to the formation of new ethers, peroxides, alcohols, carboxylic acids, and other oxygen-containing functional groups. Figure 7 shows the difference spectra for the C–O and O–H regions of PO and PO–PbO during curing at 70 °C. While it is challenging to attribute contributions in these regions to specific vibrational modes, there is clearly a much weaker increase in absorbance in the C–O and O–H regions in the presence of PbO (Figure 7b,d). It is interesting to note that, also in these spectral regions, nearly all the change in PO–PbO occurs in the first ∼2 h, which confirms previous conclusions that there is very slow chemical reactivity after the gel point. Similar trends were observed for LO, WO, SaO, and StO. The shape of the OH band envelope was also altered by the addition of PbO (Figure 7c,d). In PO, the OH band was very broad with a maximum centered at around ∼3460 cm^–1^, while in PO–PbO, the OH band was both weaker and shifted toward higher wavenumbers (∼3550 cm^–1^) with almost no absorbance below 3300 cm^–1^. One reason for the reduced OH band in PO–PbO is that the carboxylic acid concentration is lower due to lead carboxylate formation. The reduced absorbance below ∼3300 cm^–1^ can also indicate that there was a much smaller contribution of O–H groups hydrogen-bonded to other alcohols, acids, or water, which again points to a lower degree of oxidation for oil films cured with PbO.^49^

**Figure 8:**
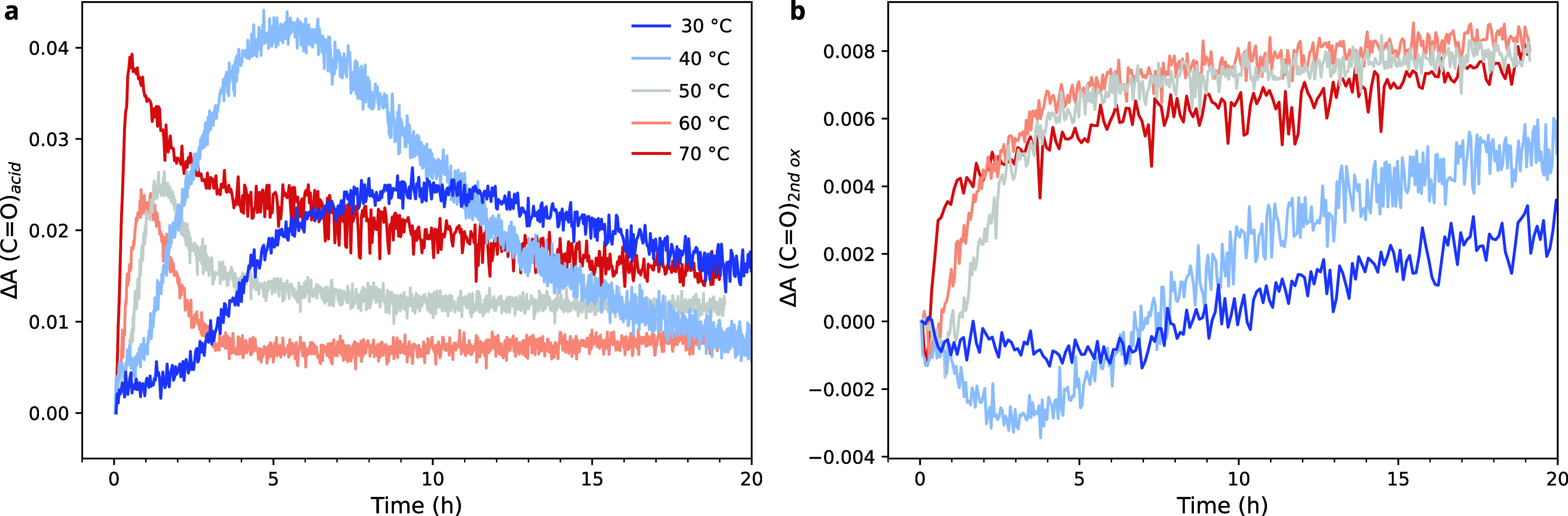
Changes in absorbance of (a) ν(C=O)_acid_ at 1695 cm^–1^ and (b) ν(C=O)_second ox_ at ∼1780 cm^–1^ in LO–PbO during curing at between 30 and 70 °C. Prior to analysis, the spectra were normalized to the initial intensity of the ν(C=O)_ester_ at 1744 cm^–1^ and baseline-corrected using a line drawn between 1850 and 1810 cm^–1^.

The relation between temperature and the formation of oxidation products appears to be rather complex, which is in line with conclusions drawn from Figure S13 that temperature may affect the relative likelihood of reaction pathways during curing. Figure 8 shows the evolution of ν(C=O)_acid_ and ν(C=O)_second ox_ in LO–PbO as a function of temperature. The profiles recorded at 50, 60, and 70 °C show a gradual deceleration of acid and secondary oxidation product formation and a consumption of carboxylic acids to form lead carboxylates at all temperatures (Figures S16 and S17). However, at 30 and 40 °C especially, the secondary oxidation products formed much more slowly than at higher temperatures. The profiles of ν(C=O)_acid_ are challenging to interpret, with an acid concentration that reaches a particularly high maximum at 30 °C despite overall slow kinetics in the system and accompanying high concentration of lead carboxylates (Figures S17 and S18). These changes in oxidation product formation with temperature suggest that the curing pathways shift to some degree to favor oxidation at temperatures in the range of 40–50 °C, though pinpointing which changes occur exactly remains challenging based on these data.

### Oil Curing at Ambient Conditions

**Figure 9:**
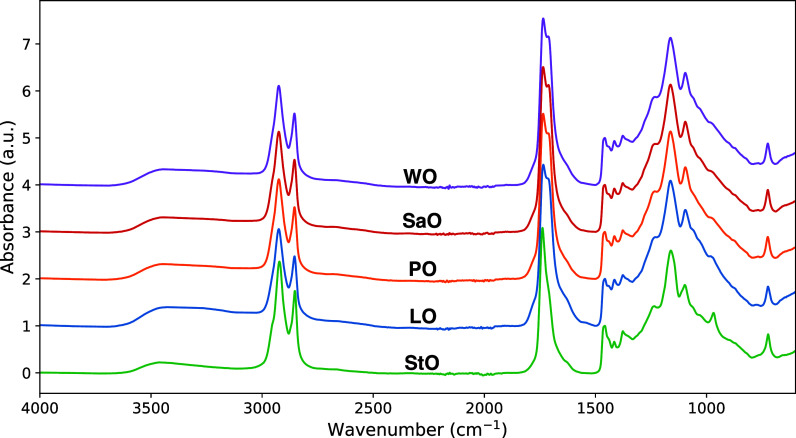
ATR-FTIR spectra of LO, WO, SaO, PO, and StO after 1 year of curing in ambient conditions. The spectra were baseline-corrected using lines between 3900, 2500, 1900, and 1500 cm^–1^ and they were normalized to the δ(CH_2_) vibration band at 1465 cm^–1^.

The data presented in Table 2 suggests that there is considerable variation in the rate of curing between the oils selected for this study and that both catalysis by PbO and temperature play a big role in these processes. Figure S11 also shows that the spectra of the oils, with and without PbO, at the end of each curing run are remarkably different, pointing to large variations in chemical composition. It is interesting to contrast these findings with an experiment in which the five oils, without PbO, were left to cure at ambient conditions (∼22 °C and 50% RH) for 1 year (Figure 9). Under those conditions, LO, WO, PO, and SaO show nearly identical ATR-FTIR spectra after 1 year, with only very minor differences in the O–H stretch region for LO, which suggests that the long-term chemical functionality is very similar for these oils. In agreement with its prepolymerization process, StO shows less intense vibrations associated with O–H, aldehyde and acid C=O, and C–O, which point to an overall lower degree of oxidation.

While these spectra provide no information on potential clustering in the network structure of these oils, they do suggest that, apart from StO, most differences occur in the chemical functionality and structure of drying oil polymers when comparing oils that have been cured with or without a catalyst or when comparing oils that have been cured at vastly different temperatures.

## Conclusions

FRP of highly functional monomers, such as autoxidation of drying oils, is an incredibly complex process. ATR-FTIR spectroscopy is a powerful tool to investigate the curing kinetics and evolving chemical structure in these systems, as it allows quantitative monitoring of key functional groups during the entire curing process with high time resolution under a wide variety of conditions.

Monitoring of the consumption of *cis* C=C bonds allowed detection of a phase transition in the curing oils, similar to a gel point, that drastically reduces the rate of curing, and both fast and slow rate parameters could be estimated. This effect is especially strong when catalytically active PbO is added to the oils. Investigation of the decrease in curing rate caused by the phase transition, in combination with the conversion of *cis* C=C bonds at the transition, supports the hypothesis that a polymer network with highly heterogeneous cross-link density is formed in the presence of PbO as a catalyst. Oil reactivity appears to be diffusion-controlled beyond the phase transition, as there is little to no temperature dependence of reactivity after that point.

Analysis of other features in the ATR-FTIR spectra of curing oils demonstrated that also C=C bond isomerization and oxidation reactions are extremely slow after the phase transition, and the presence of PbO caused lower levels of oxidation after curing. There is additional evidence that lower curing temperatures in oil mixtures with PbO favor oxidation reactions that lead to carboxylic acid formation. Despite a rather different fatty acid distribution, and variations in curing kinetics, the differences in chemical composition between LO, PO, WO, and SaO appear to be rather minor on a long time scale. An exception is StO, which stands out in all analyses with slower curing rates and relatively low levels of oxidation after curing.

The long-term consequences of heterogeneous cross-link density in oils cured with PbO are difficult to predict. However, one can imagine that the diffusion behavior of solvent, water, or other reactive molecules is rather different in heterogeneous networks,^50^ as will be the depolymerization kinetics and associated mechanical properties of the networks as a consequence of degradation reactions during aging. Both of these consequences of network heterogeneity are highly relevant in the context of oil painting conservation. Furthermore, it is interesting to highlight that the intuition that the addition of the PbO catalyst leads to faster and more extensive curing is not always true; for most oils, PbO causes a network that has *less* extensive global cross-link density and lower levels of oxidation. Additionally, the common practice of accelerating oil paint drying for research purposes by addition of curing catalysts and increasing temperature needs to be carefully considered because those choices may induce a paint structure that is dissimilar to historical oil paint in a meaningful way.

## Data Availability

All raw ATR-FTIR spectroscopy data is publicly available through an online repository: doi.org/10.21942/uva.25399810.v1.

## Supporting Information Available

Method descriptions of acid value determination by titration and fatty acid distribution determination by NMR spectroscopy, ATR-FTIR spectra, and processed data (PDF)
