# Supplementary material for: Comprehensive Characterization of Drying Oil Oxidation and Polymerization Using Time-Resolved Infrared Spectroscopy
Source: Macromolecules. 2024 Aug 28;57(17):8263–76. doi: 10.1021/acs.macromol.4c01164 (PMC11394013; doi:10.1021/acs.macromol.4c01164)
Supplement: Supplementary file 1 — ma4c01164_si_001.pdf [file ma4c01164_si_001.pdf]

Supporting Information

Comprehensive characterization of drying oil oxidation and polymerization using time-resolved infrared spectroscopy

Gwen DePolo, Piet Iedema, Kenneth Shull and Joen Hermans*

Van 't Hoff Institute for Molecular Sciences, University of Amsterdam,
PO box 94157, 1090GD Amsterdam, The Netherlands.
E-mail: j.j.hermans@uva.nl

Contents

A Methods **2**
A.1 Acid value determination by titration 2
A.1.1 Background 2
A.1.2 Titration procedure 2
A.2 Fatty acid distribution determination by NMR spectroscopy 3
A.2.1 Measurements 3
A.2.2 Calculations of fatty acid distributions 3

B Modeling overlapping IR bands **11**
B.1 Example of a deconvolution of C=C bands 11
B.2 Attempts to deconvolute the carbonyl bands in drying oils 11

C Kinetic fits to cis C=C consumption data **13**

D Additional ATR-FTIR spectroscopy data **16**

E Observations on lead carboxylate formation **20**

A Methods

A.1 Acid value determination by titration

A.1.1 Background

The acid value (AV) provides a measure of the concentration of free fatty acids (FFA) present in an oil and is typically expressed using units of mg of base required to neutralize one gram of oil (mg_{base}/g_{oil}). One of the most common ways to determine the AV is to titrate the oil with a solution of strong base. The following equation wa used to determine the AV from titration results:

$$\mathrm{AV} = \frac{(\mathrm{V_T} - \mathrm{V_B}) * \mathrm{N_T} * \mathrm{Mw_{NaOH}}}{\mathrm{m_{oil}}}, \tag{1}$$

where AV is the acid value (mg_{NaOH}/g_{oil}), V_T is the volume of titrant used to neutralize the oil sample (mL), V_B is the volume of titrant used to neutralize the ethanol solution before the addition of the oil sample (mL), N_T is the concentration of the titrant (mmol/mL), Mw_{NaOH} is the molar mass of sodium hydroxide (40 mg/mmol), and m_{oil} is the mass of the oil used for the titration (g). A similar metric %FFA expresses the concentration of FFA in a drying oil, or the weight percentage of free fatty acids in the drying oil. This value requires expression as a specific type of fatty acid and is assumed to be oleic acid for most drying oils. The equation to determine the %FFA is very similar to the equation to determine AV

$$\%\mathrm{FFA_{oleic}} = \frac{(\mathrm{V_T} - \mathrm{V_B}) * \mathrm{N_T} * \mathrm{Mw_{oleic}}}{\mathrm{m_{oil}}}, \tag{2}$$

where the molar mass of oleic acid is 282.45 mg/mmol. The following factor can be used to convert between AV and %FFA

$$\frac{\mathrm{AV}}{\%\mathrm{FFA}} = \frac{\mathrm{Mw_{NaOH}}}{\mathrm{Mw_{oleic}} \times 0.1} = 1.42. \tag{3}$$

CHECK THIS The factor of 0.1 is a result of incorporating a conversion factor of 1g/1000mg and multiplying by 100 to express the concentration of FFA as a percentage [6].

A.1.2 Titration procedure

A Metrohm 888 Titrando, 801 Stirrer, and Solvotrode easyClean non-aqueous electrode were used to perform autotitrations of the oil samples. A standard FFA reagent was prepared to add to the oil sample for the titrations using 1 L of 95% ethanol, 500 mL of toluene, 60 mL of phenolphthalein indicator, and 5 mL of 0.1 M HCl, which were mixed well before measurements. The 0.1 M NaOH titrant solution was a reagent-grade solution and the autotitrator reservoir was filled before the start of the titrations. A blank measurement was performed to determine V_B by placing 50 mL of the FFA reagent in a beaker, transferring the electrode to the solution from a distilled water beaker, and performing a titration to the endpoint as determined by a faint color change and by a jump in the potentiometric curve measured by the autotitrator. After the V_B was recorded, the electrode was rinsed with acetone and stored in the distilled water beaker. To measure the oil samples, 0.1 − 0.3 g of the oil was weighed into a beaker, then 50 mL of the FFA reagent was added. The solution was stirred well, then the electrode was inserted, the mass was recorded on the Titrando, and then the titration was performed. After the titration was complete, the autotitrator provided the $V_T$and the

Table 1: The average %FFA values for the five drying oils along with their standard deviations.

Drying oil	$\%\text{FFA}_{\text{oleic}}$
Linseed	1.18 ± 0.02
Walnut	1.08 ± 0.05
Poppyseed	0.28 ± 0.01
Safflower	0.78 ± 0.02
Stand	2.95 ± 0.04

%FFA value for the sample, which was recorded. The electrode was then rinsed with acetone and stored in the distilled water beaker. When the autotitrator did not find a clear endpoint, the electrode was rinsed or placed in a beaker of acetone to remove the oil that had gathered on the surface of the electrode. Each drying oil was measured 3-5 times, the %FFA values were averaged and the values are provided in Table 1. The %FFA values converted to AV are reported in Table 1 in the main paper.

A.2 Fatty acid distribution determination by NMR spectroscopy

A.2.1 Measurements

To prepare the samples for ^{1}H and ^{13}C spectra collection, 200 μL of the drying oil was added to 400 μL of deuterated chloroform (Sigma Aldrich), mixed well, and transferred to an NMR tube. ^{1}H-NMR and ^{13}C-NMR spectra were collected using a Bruker Avance III 500 MHz spectrometer at room temperature. 10 scans were averaged with a relaxation delay of 15 s for ^{1}H spectra and 20 s for ^{13}C. Peak integrals were calculated from the FIDs using MestReNova software. Two analysis approaches were considered: one using solely the ^{1}H data (based on the approach used in Barison *et al.*[3]) and one that combines both the ^{1}H and ^{13}C data. The second method was deemed more reliable, and is outlined below.

A.2.2 Calculations of fatty acid distributions

The goal of the calculation is to determine the mol fraction of each unsaturated fatty acid (linolenic, linoleic, and oleic acid) and saturated fatty acids (most likely palmitic and stearic acid) in each drying oil. This goal can be achieved by working with the average number of C=C bonds per fatty acid (FA) chain, $\overline{\text{C=C}}$, which can be expressed as

$$\overline{\text{C=C}} = \frac{C_{\text{ln}} n_{\text{ln}} + C_{\text{le}} n_{\text{le}} + C_{\text{ol}} n_{\text{ol}} + C_{\text{sat}} n_{\text{sat}}}{}. \tag{4}$$

In this equation, C_{ln}, C_{le}, C_{ol}, and C_{sat} are the relative concentrations of each fatty acid chain (ln = linolenic acid, le = linoleic acid, ol = oleic acid, sat = saturated fatty acids), and n is the number of C=C bonds per chain in each of the fatty acids (3, 2, 1 and 0). With this method, it is only possible to distinguish fatty acids with unique numbers of C=C bonds. For instance, we can only obtain a mol fraction of the group of saturated fatty acids (mostly palmitic and stearic acid in the case of drying oils), not individual saturated fatty acids. We can calculate the mol fraction of each fatty acid type with the following steps:

1. determine the value of $\overline{\text{C=C}}$ from ^{1}H NMR spectra;
2. determine the values of C_{ln}, C_{le}, C_{ol} from ^{13}C NMR spectra;
3. calculate the value of C_{sat} using equation 4;
4. calculate the mol fraction of each fatty acid.

As an example, we will demonstrate the calculation using the NMR data from LO as an example. The ^{1}H NMR and ^{13}C NMR spectra for LO are shown in Figure S1. For the ^{13}C NMR spectrum (Figure S1a), just the region where C=C signals are present is shown. In this region, peaks associated with linolenic acid occur at $\delta \sim 131.79$, 130.08, 128.19, 128.15, 127.71, and 127.07 ppm, peaks associated with linoleic acid occur at $\delta \sim 129.9$, 129.83, 128.01, and 127.85 ppm, and the peaks associated with oleic acid occur at $\delta \sim 129.86$ and 129.6 ppm[1, 2, 5]. In the ^{1}H NMR spectrum (Figure S1b), the relevant peaks are centered at $\delta \sim 5.29$ppm (C=C−$\underline{\text{H}}$) and $\delta \sim 0.82 - 0.93$ ppm (C$\underline{\text{H}_3}$)[3].

Step 1 The value of $\overline{\text{C=C}}$ can be determined from ^{1}H NMR spectra by calculating integrals for the C=C − $\underline{\text{H}}$ and the C − $\underline{\text{H}_3}$ proton peaks and dividing by the number of protons contributing to each peak.

$$\underline{\text{H}} - \text{C=C} - \underline{\text{H}} : \frac{6.80}{2} = 3.4 \tag{5}$$

which represents the number of C=C bonds in the oil.

$$\text{C}\underline{\text{H}_3} : \frac{4.75}{3} = 1.583 \tag{6}$$

which represents the total number of fatty acid chains in the oil. Dividing these two values yields the average number of C=C bonds per FA chain:

$$\overline{\text{C=C}} = \frac{3.4}{1.583} = 2.147 \tag{7}$$

Step 2 We can determine the values of C_{ln}, C_{le}, C_{ol} from ^{13}C NMR spectra by summing the relevant C=C carbon peak integrals and dividing by the number of unsaturated carbons for the specific fatty acid. For linolenic, linoleic, and oleic acids, the values would be:

$$C_{\text{ln}} = \frac{1 + 0.68 + 2.08^{*} + 0.68 + 1}{6} = 0.91; \; * = \text{combined area for 2 peaks} \tag{8}$$

$$C_{\text{le}} = \frac{0.53 + 0.26 + 0.26 + 0.27}{4} = 0.33 \tag{9}$$

$$C_{\text{ol}} = \frac{0.3 + 0.33}{2} = 0.315 \tag{10}$$

Step 3 We can fill in the calculated values in Eq. 4, and solve for C_{sat}:

$$\overline{\text{C=C}} = \frac{0.91 \times 3 + 0.33 \times 2 + 0.315 \times 1}{0.91 + 0.33 + 0.315 + C_{\text{sat}}} = 2.147 \tag{11}$$

which yields a value for $C_{\text{sat}} = 0.17$.

Step 4 The mol fraction x_{FA} (in %) of each fatty acid can be calculated with the ratios:

$$x_{\mathrm{FA}} = \frac{C_{\mathrm{FA}}}{C_{\mathrm{ln}} + C_{\mathrm{le}} + C_{\mathrm{ol}} + C_{\mathrm{sat}}} \times 100 \quad (12)$$

For LO, this calculation yields $x_{\mathrm{Ln}} = 53\%$, $x_{\mathrm{Le}} = 19\%$, $x_{\mathrm{Ol}} = 18\%$ and $x_{\mathrm{sat}} = 9.8\%$. Values for other oils can be found in Table 1 in the main text.

Figures S2-S4 provide the NMR spectra for WO, PO, and SaO. The procedures for determining the fatty acid distribution are the same as outlined above for LO. In the PO and SaO samples, no peaks corresponding to linolenic acid were observed.

For StO (Figure S5), the fatty acid distribution cannot be calculated. The StO sample is pre-polymerized, which means that many of the C=C bonds have been consumed or isomerized, leading to much more complicated C=C region in the ^{13}C NMR spectrum that cannot be straighforwardly linked to the initial fatty acid distribution.

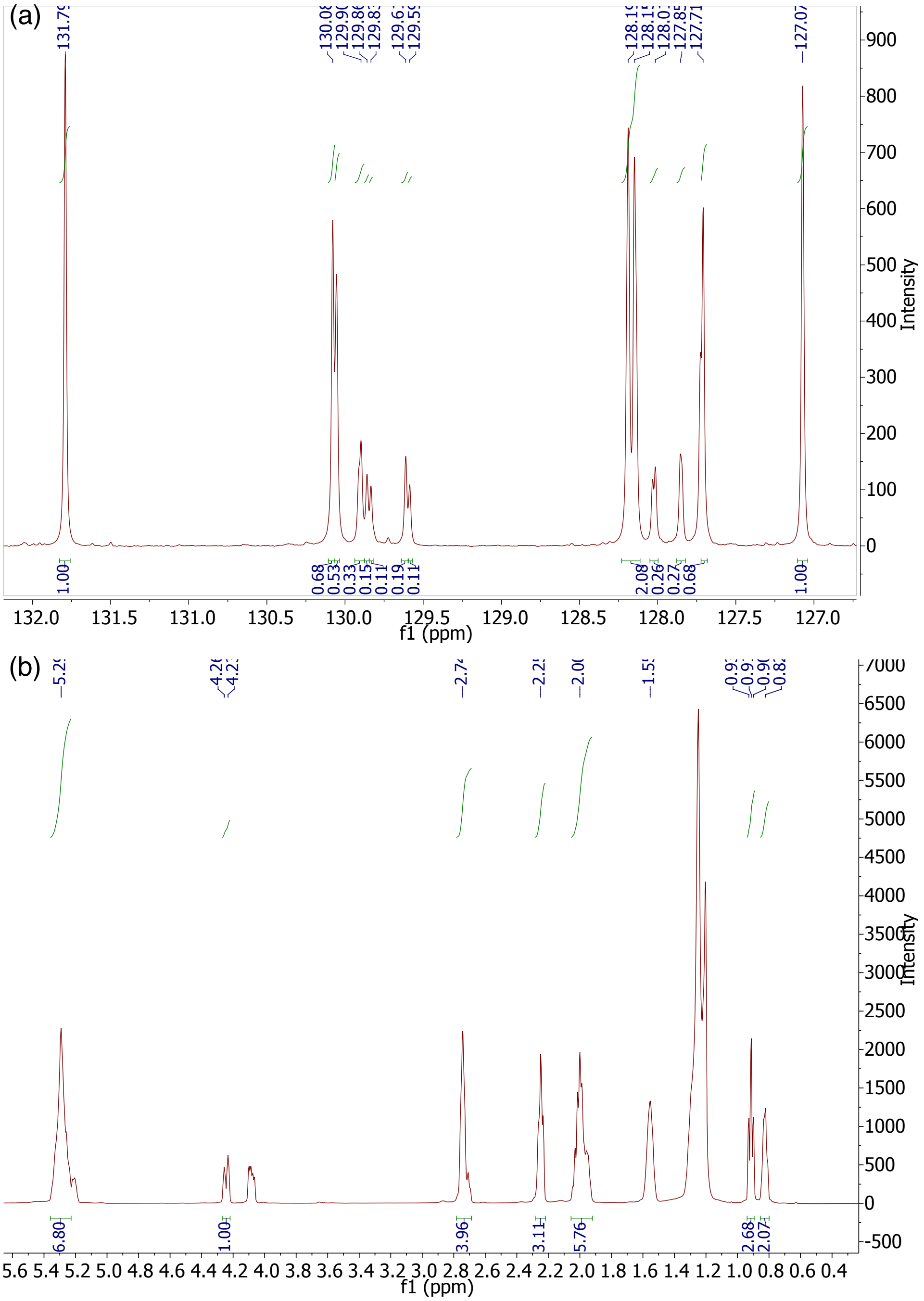

Figure S1: The (a) ^{13}C-NMR and (b) ^{1}H NMR spectra for linseed oil. The ^{13}C NMR spectrum is zoomed in to show the region of $C = C$ contributions from 127-132 ppm. Peak integrals (below spectra) and peak positions (above spectra) are provided for the peaks relevant for determining fatty acid distributions.

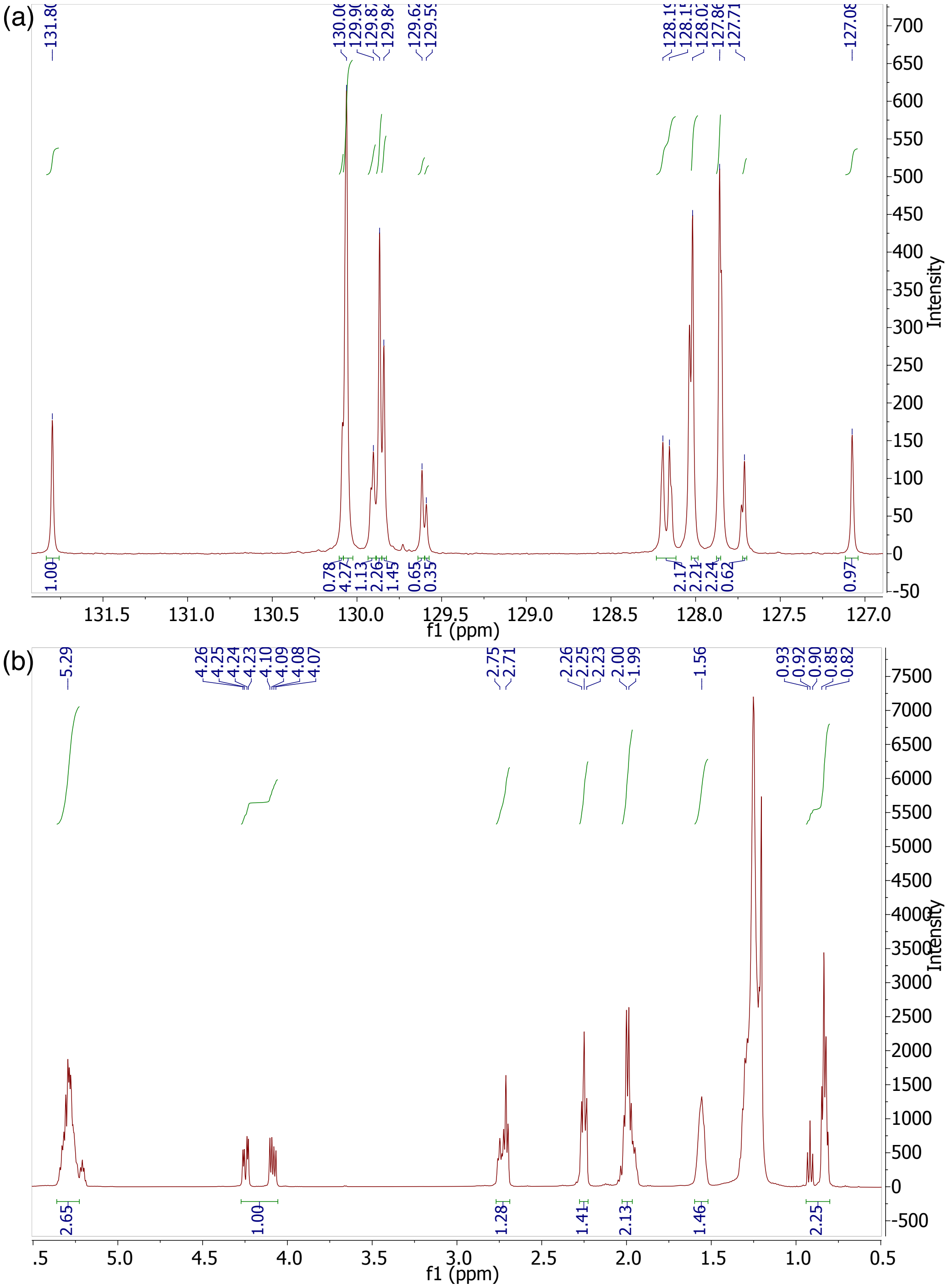

Figure S2: The (a) ^{13}C-NMR and (b) ^{1}H NMR spectra for walnut oil. The ^{13}C NMR spectrum is zoomed in to show the region of C = C contributions from 127-132 ppm. Peak integrals (below spectra) and peak positions (above spectra) are provided for the peaks relevant for determining fatty acid distributions.

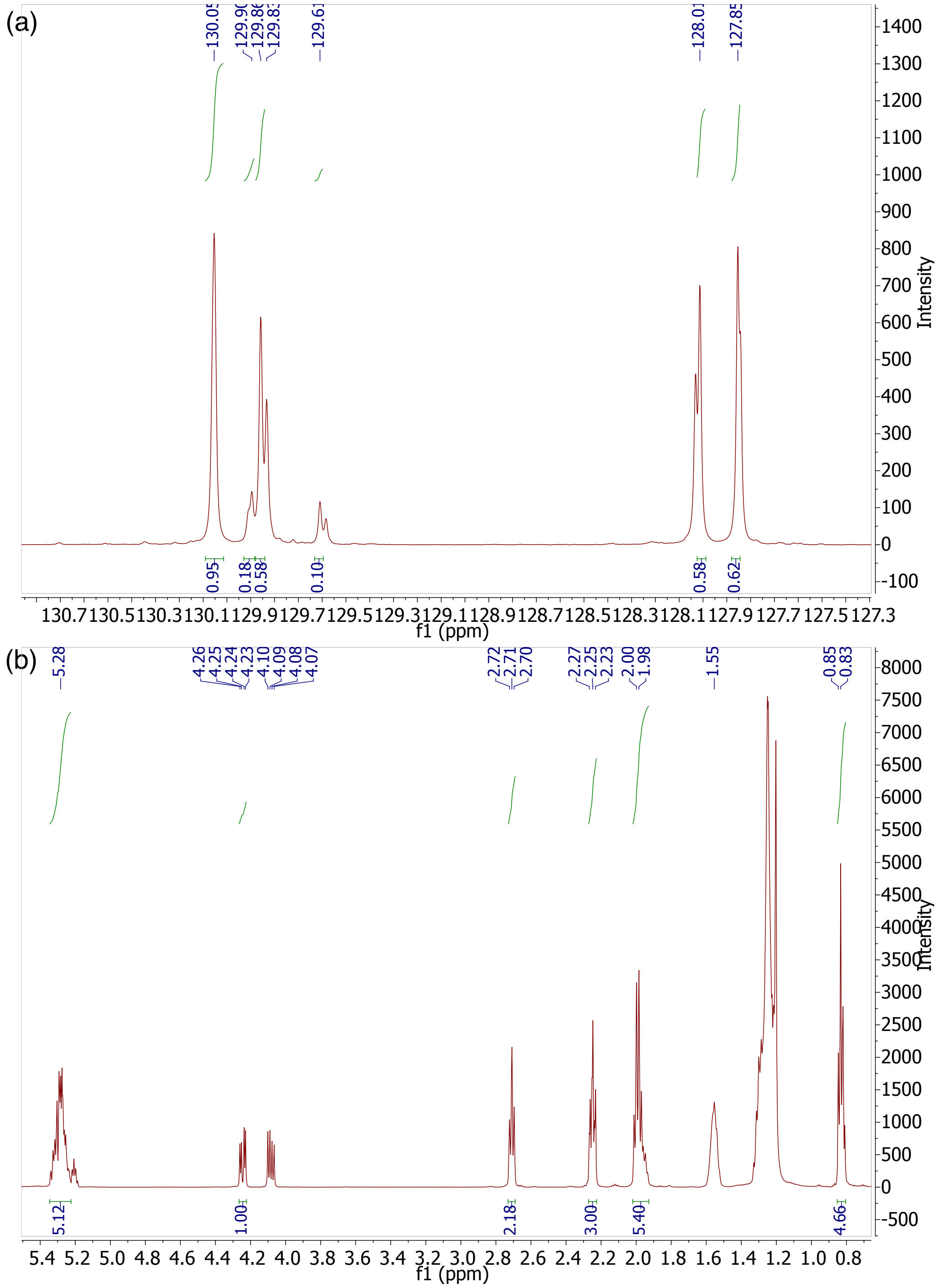

Figure S3: The (a) ^{13}C-NMR and (b) ^{1}H NMR spectra for poppyseed oil. The ^{13}C NMR spectrum is zoomed in to show the region of $C = C$ contributions from 127-132 ppm. Peak integrals (below spectra) and peak positions (above spectra) are provided for the peaks relevant for determining fatty acid distributions.

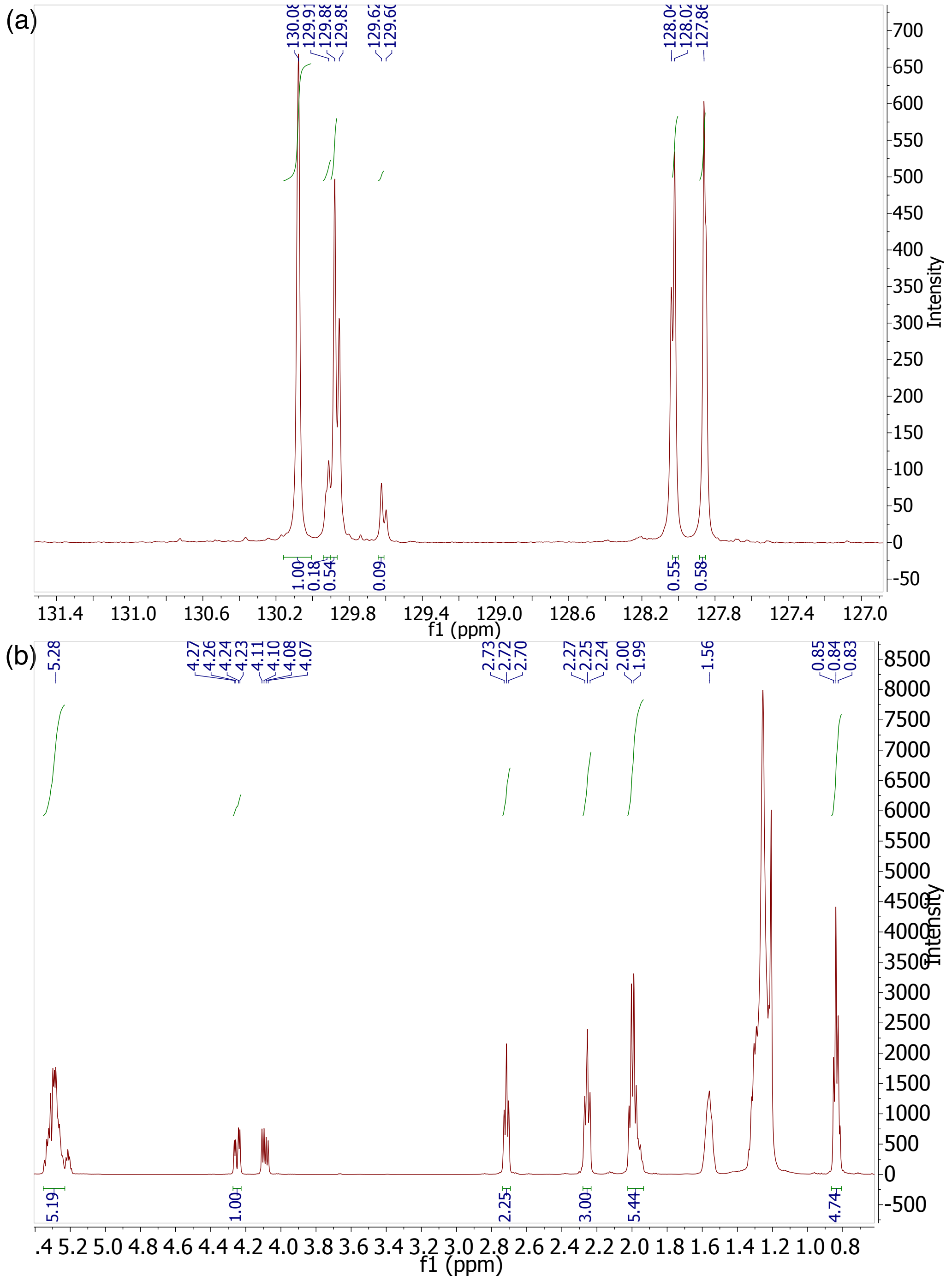

Figure S4: The (a) ^{13}C-NMR and (b) ^{1}H NMR spectra for safflower oil. The ^{13}C NMR spectrum is zoomed in to show the region of C = C contributions from 127-132 ppm. Peak integrals (below spectra) and peak positions (above spectra) are provided for the peaks relevant for determining fatty acid distributions.

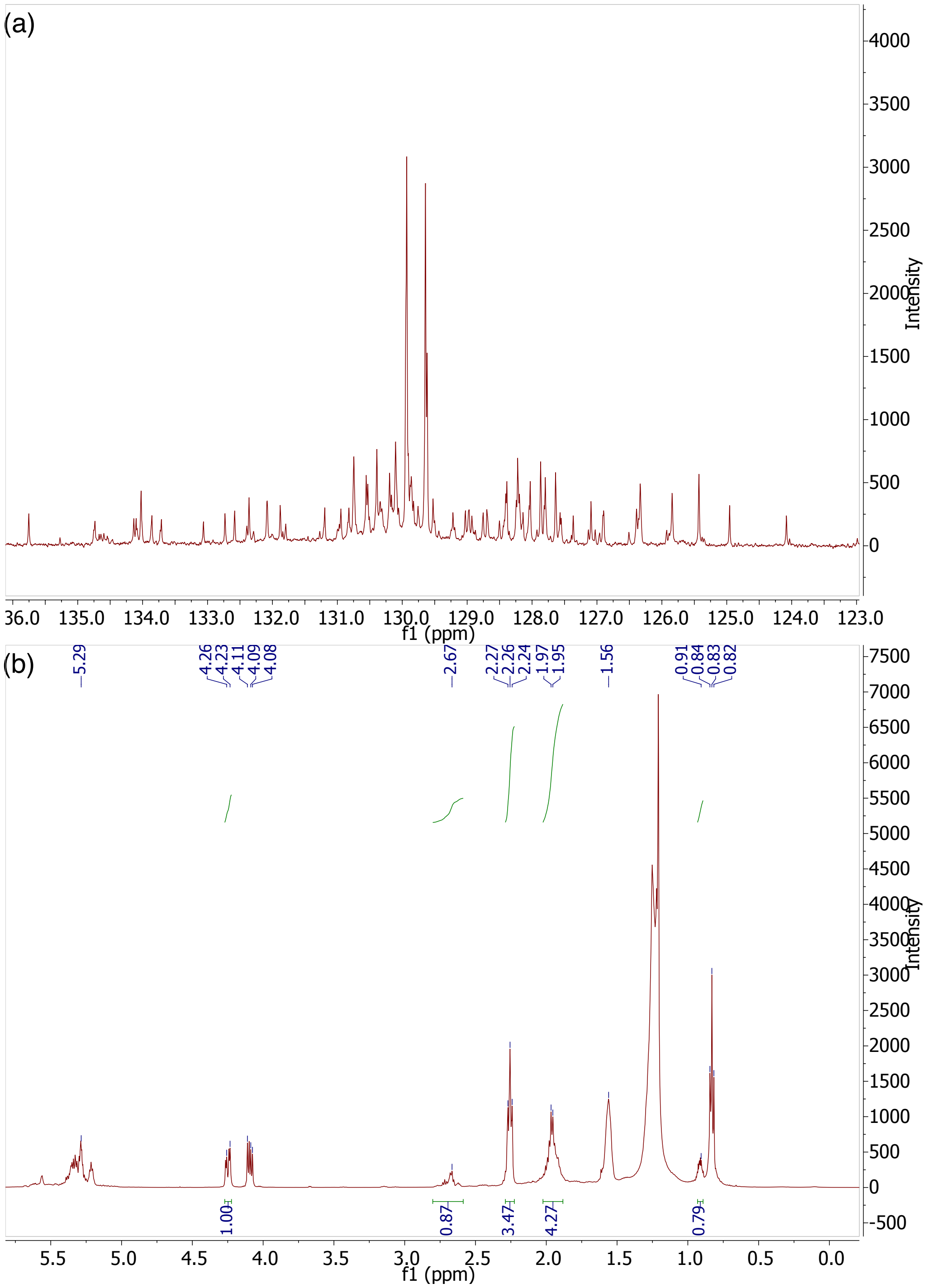

Figure S5: The (a) ^{13}C-NMR and (b) ^{1}H NMR spectra for stand oil. The ^{13}C NMR spectrum is zoomed in to show the region of $C = C$ contributions from 123-136 ppm. Due to prepolymerization of stand oil, attributing peaks in these spectra is not straightforward, and calculation is fatty acid distributions is not possible.

B Modeling overlapping IR bands

B.1 Example of a deconvolution of C=C bands

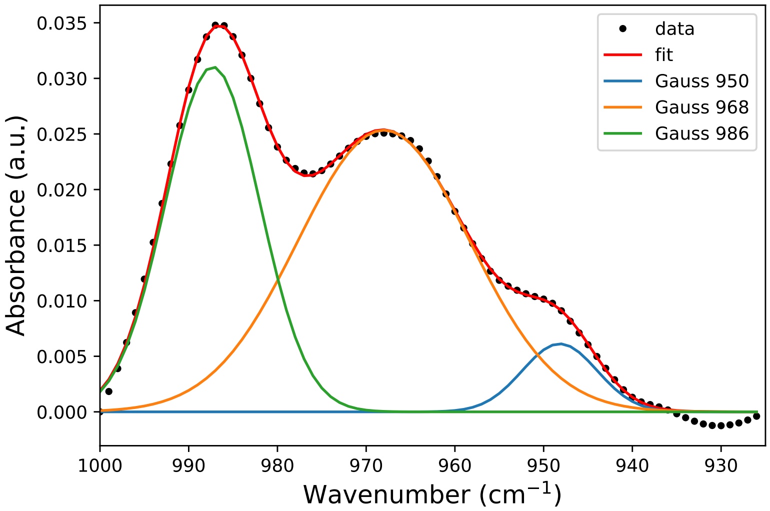

Figure S6: An example of using a linear combination of three Gaussian functions to deconvolve the individual contributions of the ω(CH) vibration bands between 900-1000 cm^{-1}. This particular spectrum corresponds to PO cured at 70 °C for ~26 h. The spectrum shown was baseline-corrected between 925 and 1000 cm^{-1}. The band at 986 cm^{-1} is attributed to ω(conjugated *trans-trans* C=C−H), at 968 cm^{-1} is attributed to the ω(non-conjugated *trans* C=C−H), and at 950 cm^{-1} is attributed to ω(conjugated *trans-cis*C=C−H).

B.2 Attempts to deconvolute the carbonyl bands in drying oils

Drawing on the successful results of deconvoluting the 900-1000 cm^{-1} region of the ω(C=C−H) with a linear combination of Guassian band shapes, an attempt was made to perform a similar analysis of the carbonyl region. The second derivative spectra of the drying oils was used to determine the most prominent contributions to the carbonyl band envelope (Figure S14), which resulted in a total of five ν(C=O) bands for the region between 1850 and 1600 cm^{-1}: 1780 cm^{-1} (secondary oxidation products), 1744-1740 cm^{-1} (ester), 1718 cm^{-1} (aldehyde), 1695 cm^{-1} (acid conjugated with a C=C bond), and 1650-1630 cm^{-1} (stretching mode of C=C transitioning from non-conjugated to conjugated). Figure S7 shows an example of the results for a least-squares fit of Gaussian band shapes to spectra recorded of LO during curing at 70 °C. As demonstrated in Figure S7a, there appears to be good agreement between the model (red curve) and the experimental data (black dots). However, when band areas are plotted over time in Figure S7b, it is immediately clear that there are two or more minimal solutions to be found with this approach that have very similar quality, leading to discontinuous jumps in the band areas. Attempts to constrain the fit further by fixing band widths was not possible without yielding other undesirable artefacts.

One major obstacle to deconvolute the carbonyl region in FTIR spectra of curing oils is that there are potentially many ν(C=O) species that contribute to the broad band beyond the ones we could include with confidence (e.g. saturated/unsaturated ketones or non-conjugated carboxylic acids).

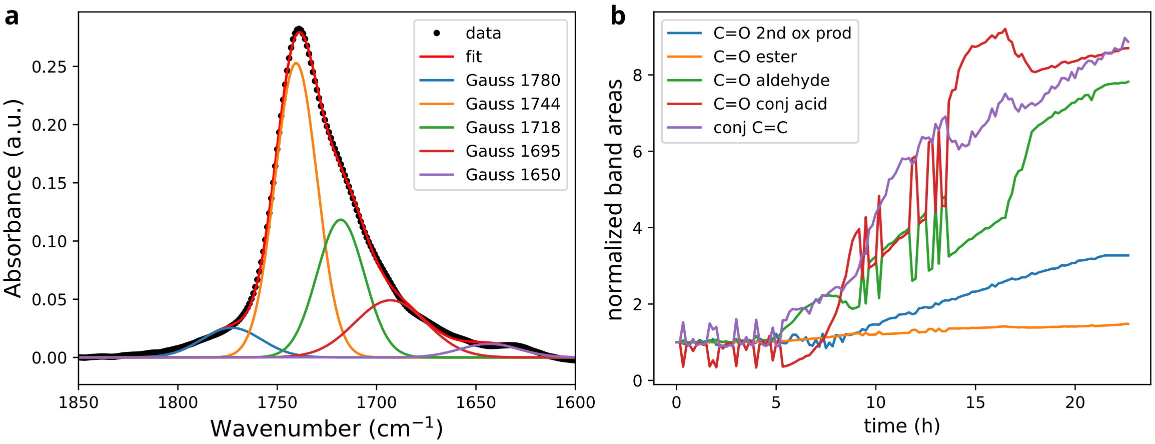

Figure S7: Band deconvolution in the carbonyl region in LO using five Gaussian band shapes. (a) An example of the result of a fit to the experimental ATR-FTIR spectrum measured at ∼20 h. (b) The band profiles of each contribution to the carbonyl band over time. The areas were normalized to the initial area of each band at $t = 0$ h.

Moreover, the center frequencies of a ν(C=O) bands may also shift by a few wavenumbers or broaden considerably as the oil transitions from a liquid film to a solid network polymer. These changes in the bands prevent the placement of very tight constraints on the fitting model, but allowing more degrees of freedom quickly creates the possibility of non-unique solutions. In this study, a more simple route of analysis of the carbonyl bands was chosen to avoid these issues, as discussed in the main article.

C Kinetic fits to cis C=C consumption data

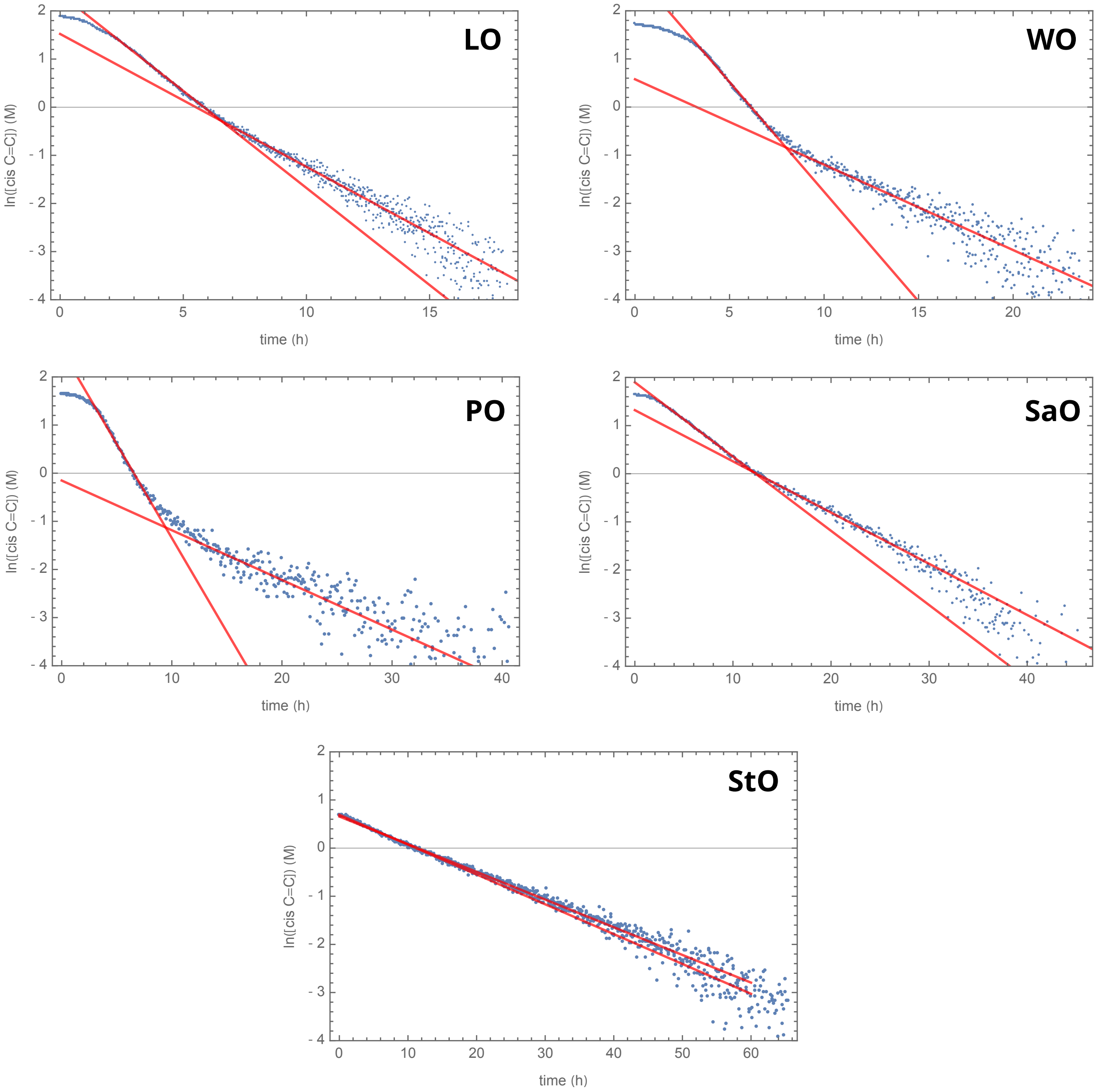

Figure S8: Best fits of two lines to the linear domains in *cis* C=C concentration data during curing at 70 °C for five oils. The slopes of the lines provide rate constants of the fast and slow phases of curing, while the intersect provides the C=C conversion at the gel point.

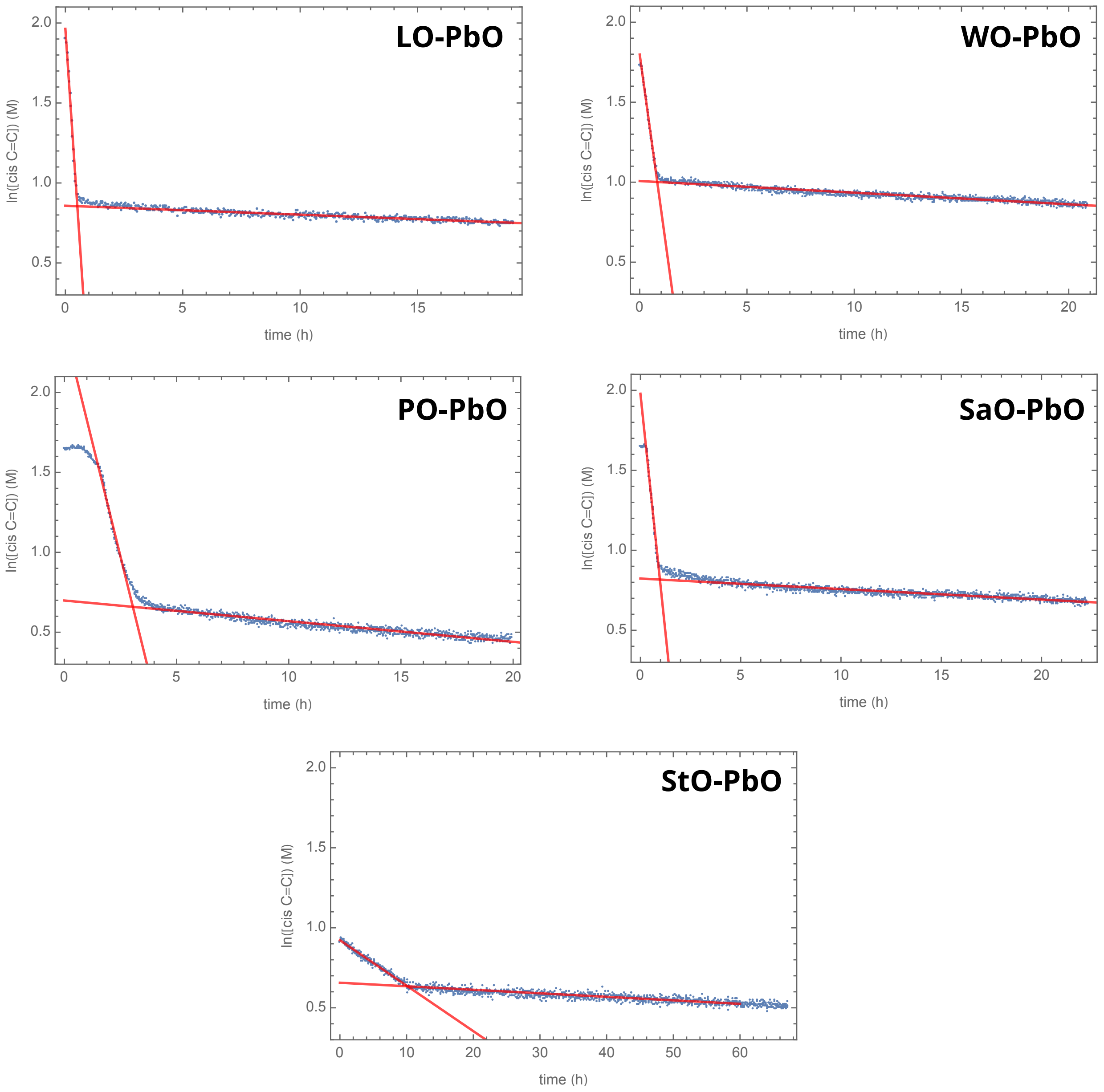

Figure S9: Best fits of two lines to the linear domains in *cis* C=C concentration data during curing at 70 °C for five oils mixed with 5 wt% PbO. The slopes of the lines provide rate constants of the fast and slow phases of curing, while the intersect provides the C=C conversion at the gel point.

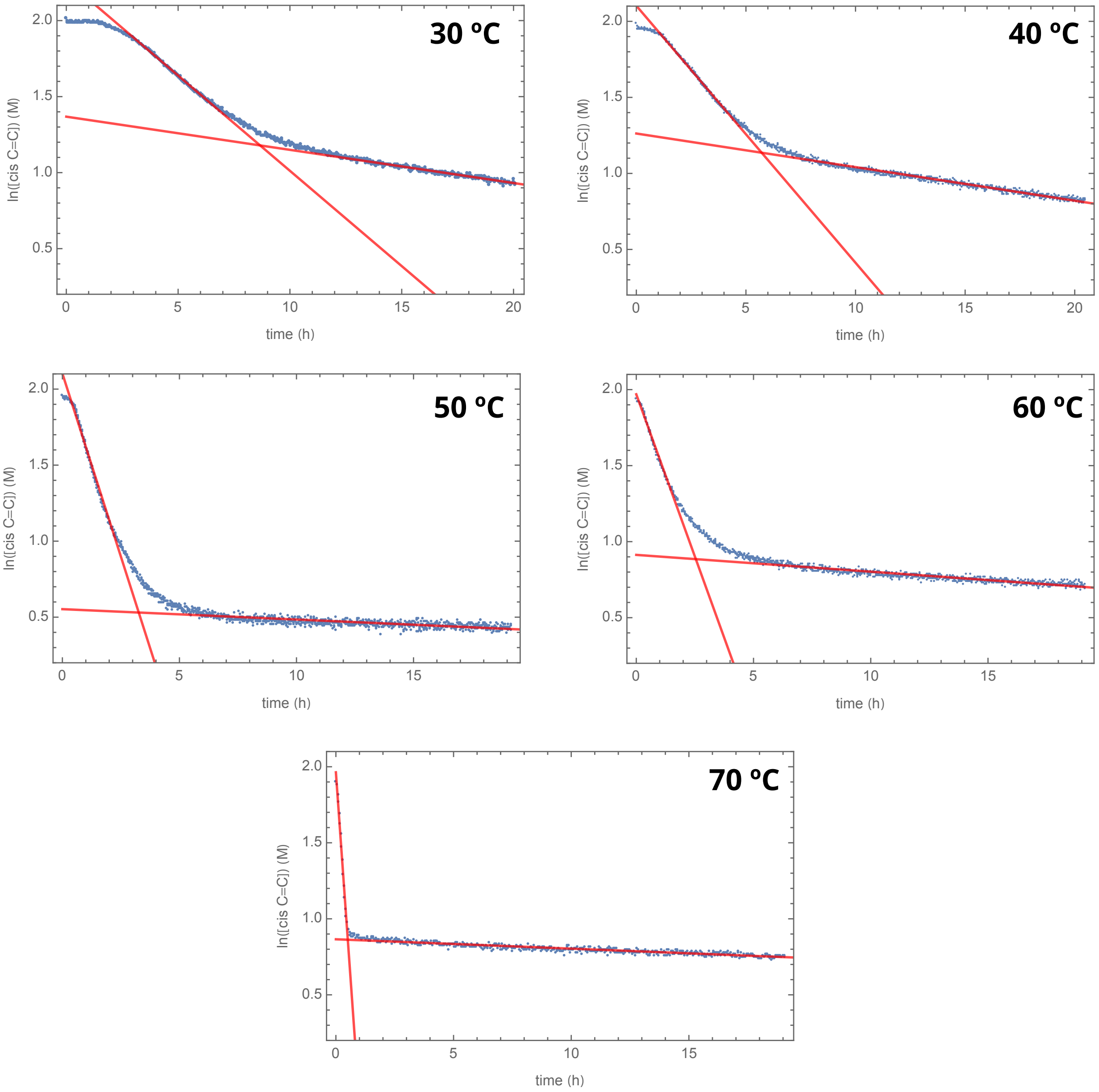

Figure S10: Best fits of two lines to the linear domains in *cis* C=C concentration data during curing of LO with 5 wt% PbO at five temperatures. The slopes of the lines provide rate constants of the fast and slow phases of curing, while the intersect provides the C=C conversion at the gel point.

D Additional ATR-FTIR spectroscopy data

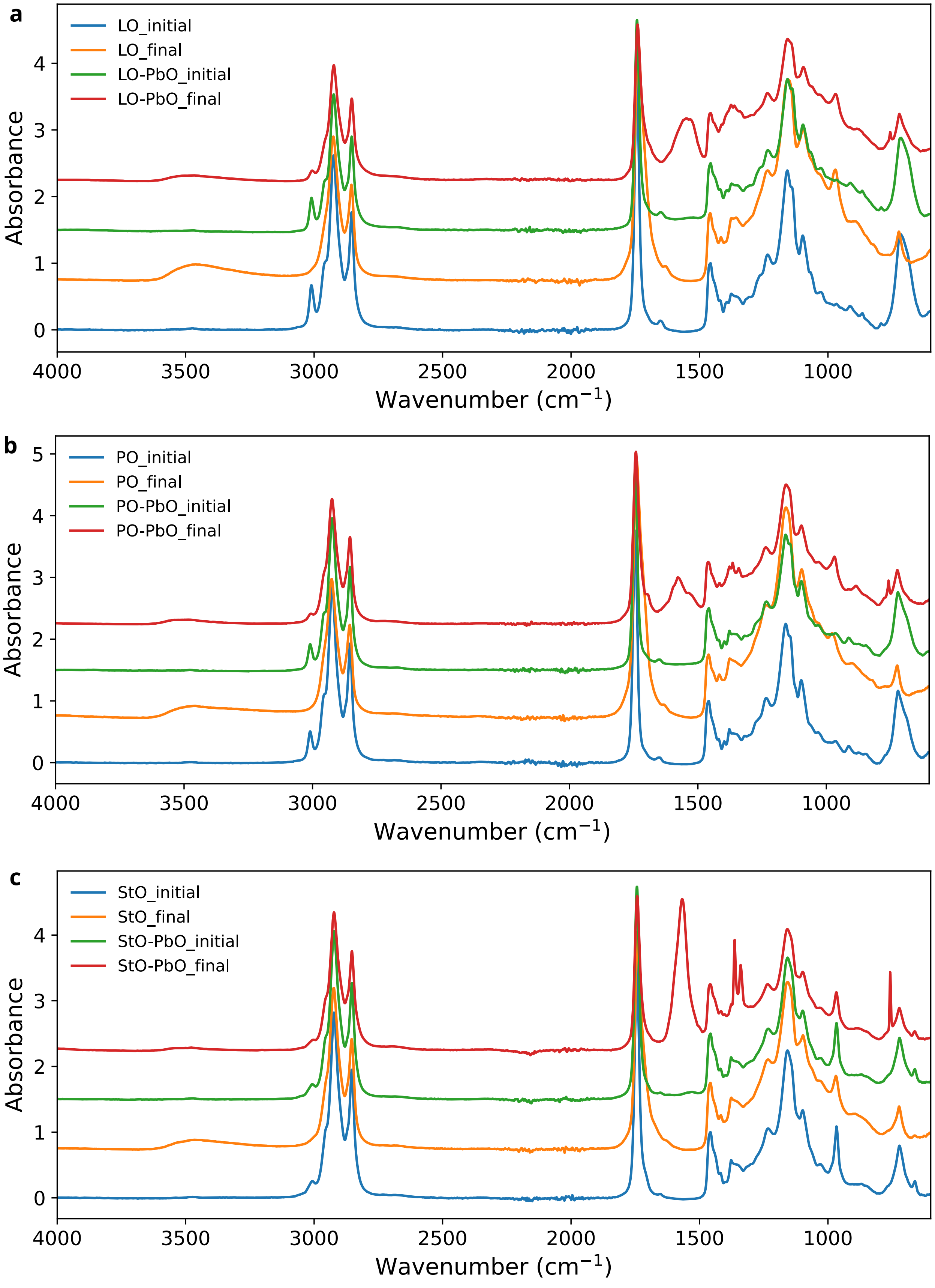

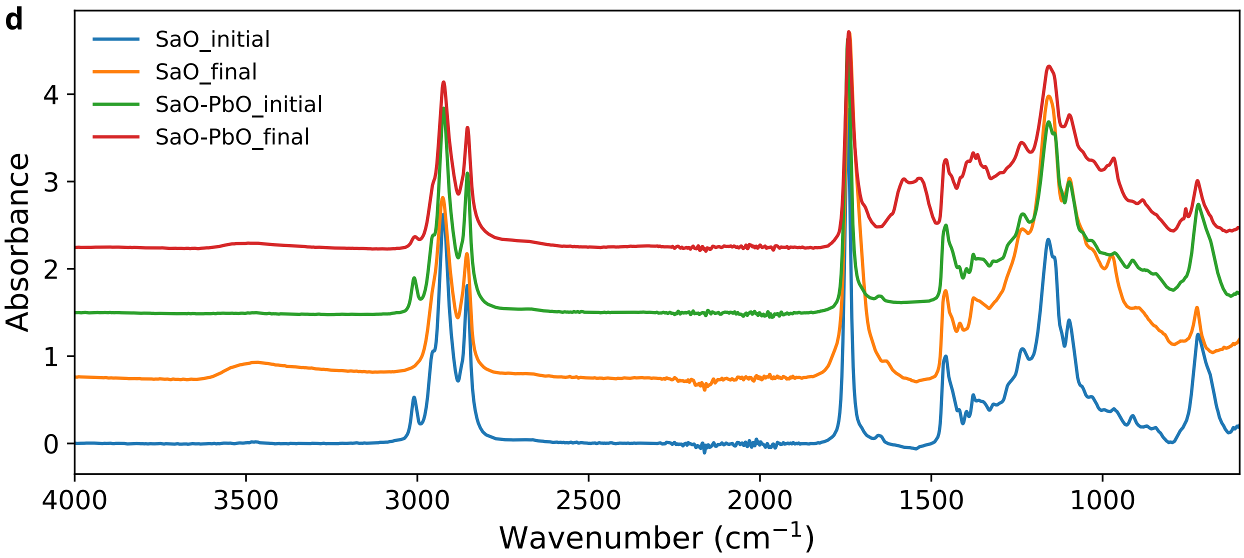

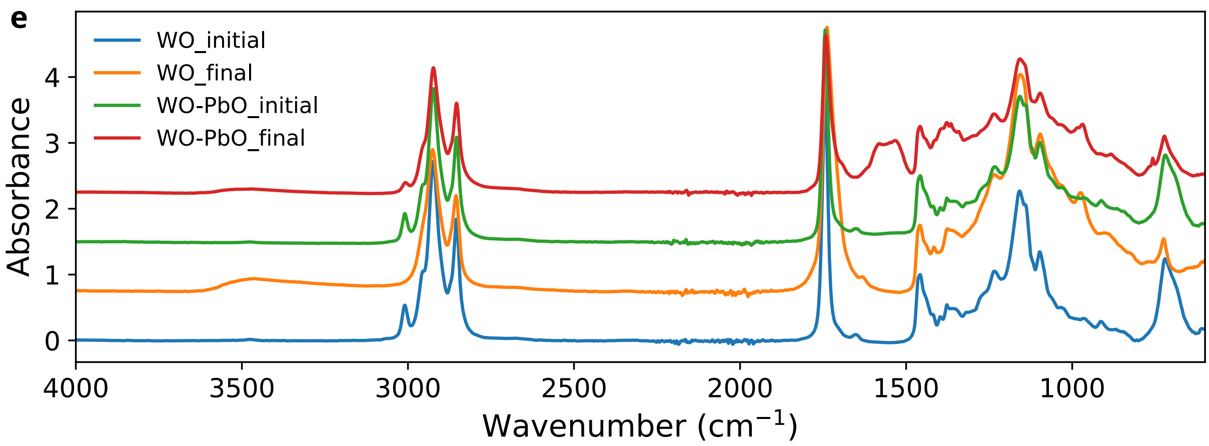

Figure S11: Initial and final spectra for the oils and oil mixtures with PbO that were cured at 70 °C. The spectra for the oils were baseline-corrected using straight lines between 3900, 2500, 1900, and 1500 cm^{-1}, while the oil mixtures with PbO were baseline-corrected with straight lines between 3900, 2500, and 900 cm^{-1}. All spectra were normalized to the δCH_2) vibration band associated with the fatty acid chains at 1465 cm^{-1}.

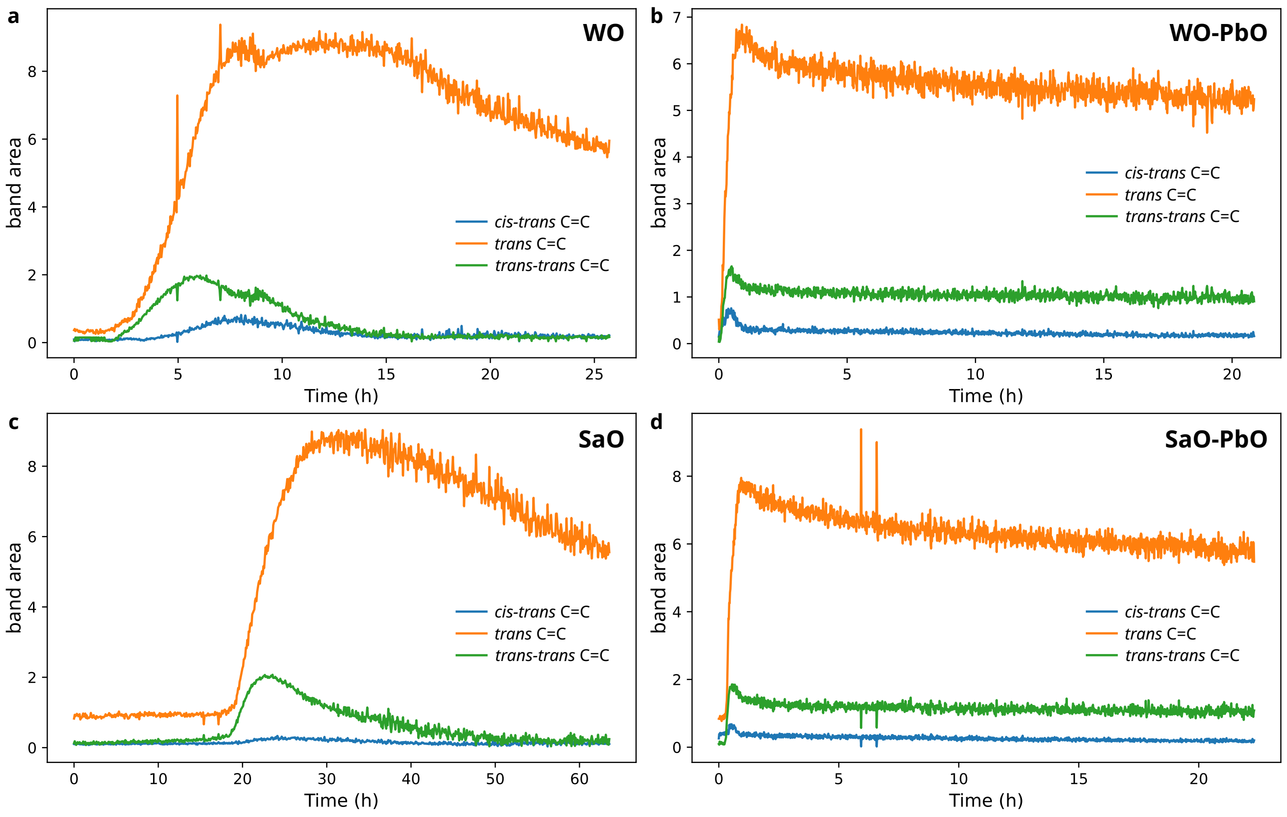

Figure S12: Comparison of the band areas corresponding to ω(CH) bands at 950 cm^{-1} (*cis-trans* C=C), 968 cm^{-1} (*trans* C=C), and 986 cm^{-1} (*trans-trans* C=C). The band areas are shown for (a) WO, (b) WO-PbO, (c) SaO and (d) SaO-PbO, all measured at 70 °C.

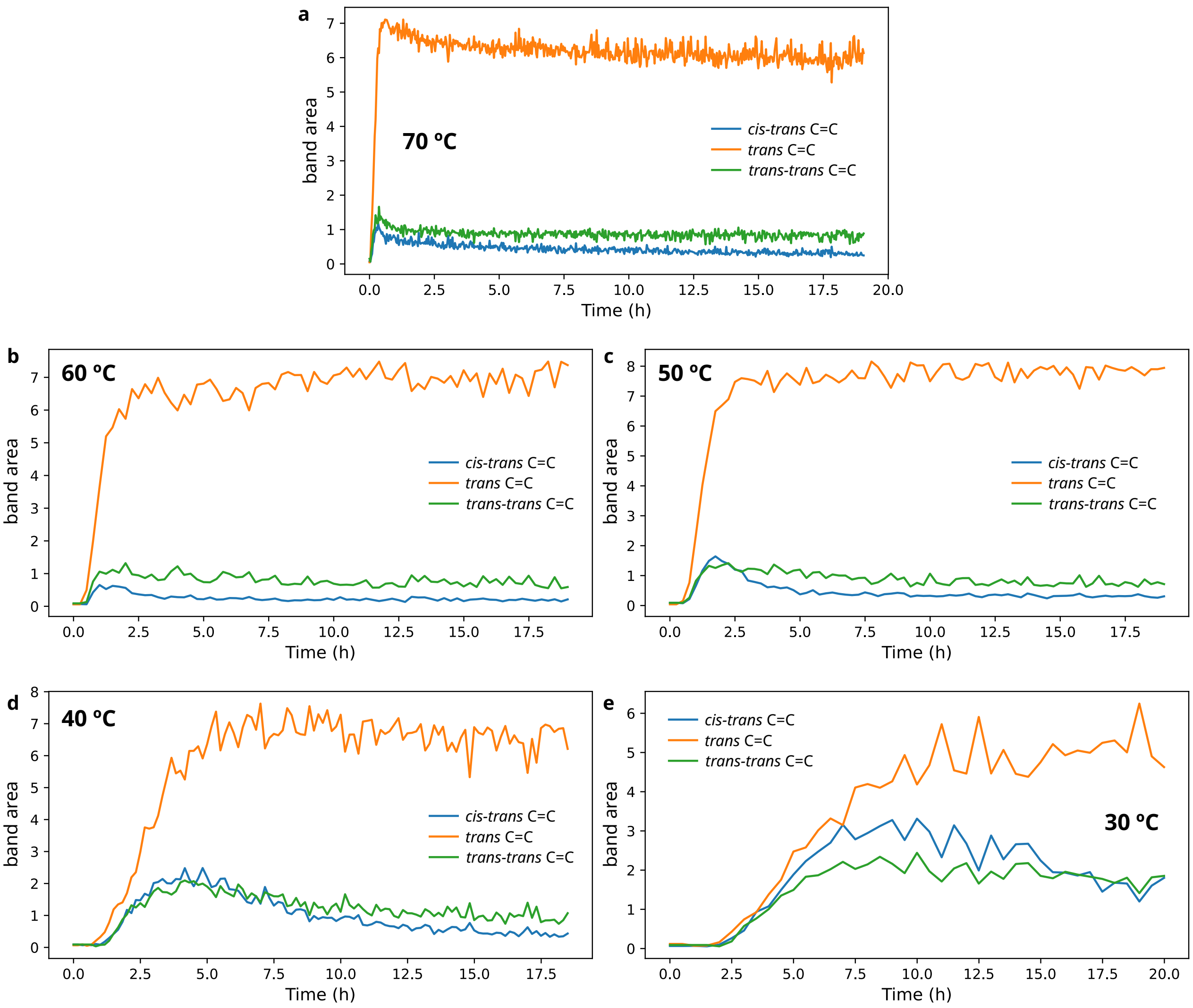

Figure S13: Comparison of the band areas corresponding to ω(CH) bands at 950 cm^{-1} (*cis-trans* C=C), 968 cm^{-1} (*trans* C=C), and 986 cm^{-1} (*trans-trans* C=C) in LO with 5 wt% PbO. The band areas are shown at (a) 70 °C, (b) 60 °C, (c) 50 °C, (d) 40 °C and (e) 30 °C. For plots (b)-(e), only every tenth spectrum was analyzed to reduce computation times.

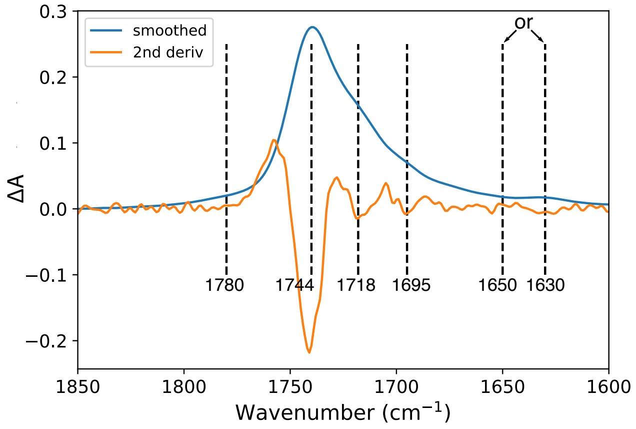

Figure S14: The carbonyl band (blue curve) and second derivative (orange curve) spectrum of LO at 17 h of curing at 70 °C. Contributing band positions are indicated with black dashed lines. The lowest wavenumber band can be either at 1650 or 1630 cm^{-1} for early and later times of cure, respectively.

E Observations on lead carboxylate formation

Lead carboxylate features were detected in ATR-FTIR spectra of oils cured with PbO as a result of reactions with carboxylic acid groups formed through oxidation and hydrolysis. Figure S15 shows the spectral region associated with asymmetric carboxylate stretch vibrations for the five oils mixed with PbO during curing at 70 °C.

There is a puzzling degree of variation in both the apparent number of bands contributing to the broad lead carboxylate feature, and even their positions. The spectra suggest bands at approximately 1620, 1595, 1585, 1570, 1555 and 1525 cm^{-1}, depending on the oil type and curing time. Some of these features have been reported before,[4] and it is known that the positions of lead carboxylate bands can shift depending on the level of order in lead carboxylate coordination.[8] However, these known reports on lead carboxylate formation do not explain the variations observed betwen the oils, for instance the appearance of a rather broad carboxylate feature for LO-PbO (Figure S15a) and a sharp feature with several distinct maxima that appear at longer curing times for PO-PbO (Figure S15c). One particular noteworthy observation is the formation of a very intense lead carboxylate band centered around 1570 cm^{-1} in StO-PbO sample (Figure S15e), which was accompanied by a doublet at approximately 1339 and 1363 cm^{-1}. This doublet, weaker versions of which were also observed in other oils, is perhaps pointing to the formation of lead formate[9, 7]. However, attempts to reproduce this observation in StO-PbO in order to carry out X-ray diffraction analysis were unsuccessful, so we are not confident about this interpretation or hypotheses why StO seems to promote the formation of formate ions.

Finally, Figure S16 shows the growth of lead carboxylate features as a function of temperature. The spectra indicate that, despite slower *cis* C=C consumption at lower temperatures, the concentration of lead carboxylates actually seems to reaches higher. A similar effect was observed for PO-PbO mixtures (Figure S17). This observation is in line with Figure 8 in the main text which showed that carboxylic acid generation seems to increase at lower temperatures, and confirms that temperature alters the relative likelihood of oxidation and crosslinking pathways during curing.

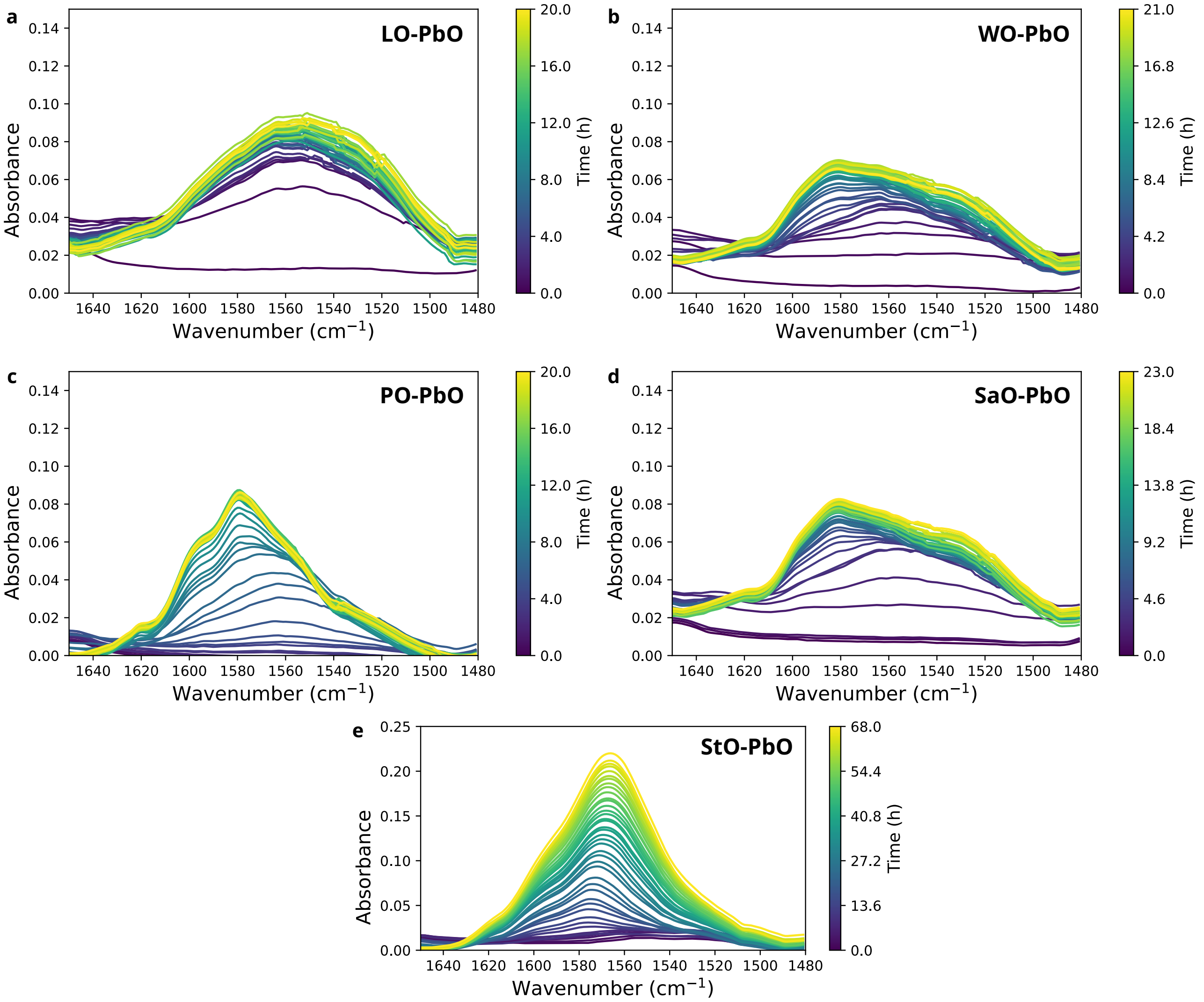

Figure S15: ATR-FTIR spectra of the lead carboxylate regions over time of cure for (a) LO-PbO, (b) WO-PbO, (c) PO-PbO, (d) SaO-PbO and (e) StO-PbO, all measured at 70 °C. To account for the tilting baseline in the raw spectra, a linear baseline between 1810 and 1850 cm^{-1} was subtracted from all spectra.

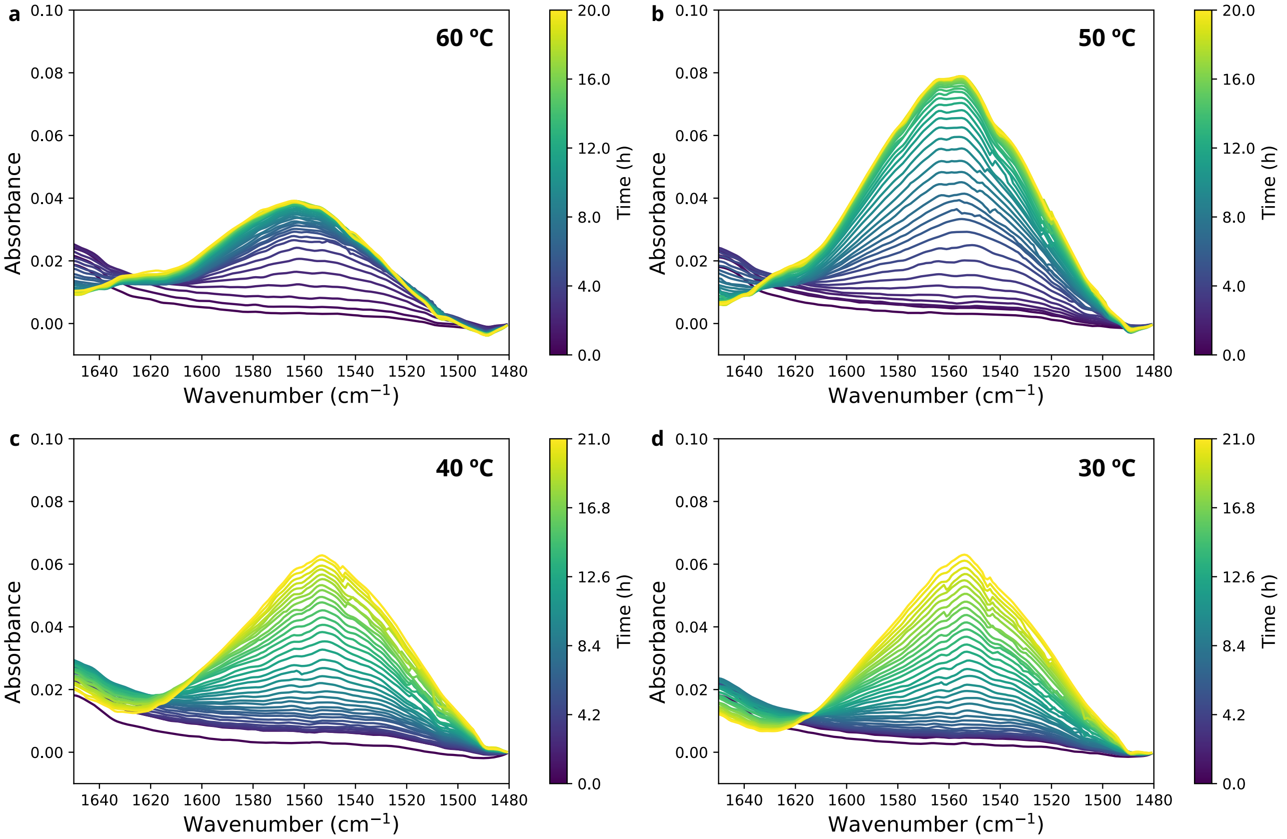

Figure S16: ATR-FTIR spectra of the lead carboxylate regions over time of cure for LO-PbO recorded at (a) 60 °C, (b) 50 °C, (c) 40 °C and (d) 30 °C. The absorbance at 1850 cm^{-1} was subtracted from all spectra as a baseline correction.

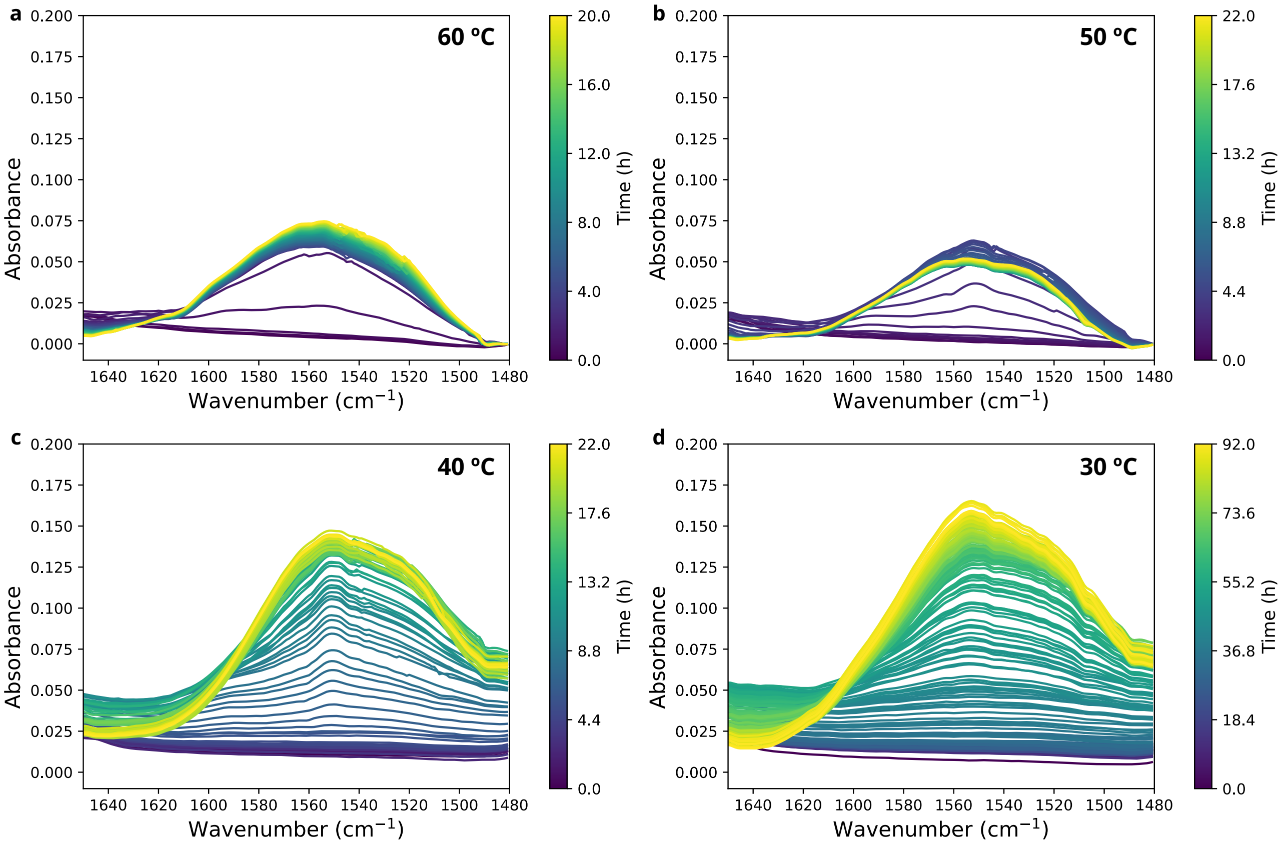

Figure S17: ATR-FTIR spectra of the lead carboxylate regions over time of cure for PO-PbO recorded at (a) 60 °C, (b) 50 °C, (c) 40 °C and (d) 30 °C. The absorbance at 1850 cm^{-1} was subtracted from all spectra as a baseline correction.

References

[1] AIST:Spectral Database for Organic Compounds,SDBS. https://sdbs.db.aist.go.jp/sdbs/cgi-bin/direct_frame_top.cgi, .

[2] AIST:Spectral Database for Organic Compounds,SDBS. https://sdbs.db.aist.go.jp/sdbs/cgi-bin/direct_frame_top.cgi, .

[3] Andersson Barison, Caroline Werner Pereira da Silva, Francinete Ramos Campos, Fabio Simonelli, Cesar Antonio Lenz, and Antonio Gilberto Ferreira. A simple methodology for the determination of fatty acid composition in edible oils through 1H NMR spectroscopy. *Magnetic Resonance in Chemistry*, 48(8):642–650, 2010. ISSN 1097-458X. doi: 10.1002/mrc.2629.

[4] L. de Viguerie, P. A. Payard, E. Portero, Ph. Walter, and M. Cotte. The drying of linseed oil investigated by Fourier transform infrared spectroscopy: Historical recipes and influence of lead compounds. *Progress in Organic Coatings*, 93:46–60, April 2016. ISSN 0300-9440. doi: 10.1016/j.porgcoat.2015.12.010.

[5] Maritza F. Díaz and José A. Gavín. Characterization by NMR of Ozonized methyl linoleate. *J. Braz. Chem. Soc.*, 18(3):513–518, 2007. ISSN 0103-5053. doi: 10.1590/S0103-50532007000300003.

[6] A. J. Dijkstra. Vegetable Oils: Composition and Analysis. In Benjamin Caballero, Paul M. Finglas, and Fidel Toldrá, editors, *Encyclopedia of Food and Health*, pages 357–364. Academic Press, Oxford, January 2016. ISBN 978-0-12-384953-3. doi: 10.1016/B978-0-12-384947-2.00708-X.

[7] Victor Gonzalez, Ida Fazlic, Marine Cotte, Frederik Vanmeert, Arthur Gestels, Steven De Meyer, Fréderique Broers, Joen Hermans, Annelies van Loon, Koen Janssens, Petria Noble, and Katrien Keune. Lead(II) Formate in Rembrandt's Night Watch: Detection and Distribution from the Macro- to the Micro-scale. *Angewandte Chemie*, 135(16):e202216478, 2023. ISSN 1521-3757. doi: 10.1002/anie.202216478.

[8] Joen Hermans, Lonneke Zuidgeest, Piet Iedema, Sander Woutersen, and Katrien Keune. The kinetics of metal soap crystallization in oil polymers. *Phys Chem Chem Phys*, 23:22589–22600, 2021. doi: 10.1039/d1cp03479k.

[9] Silvie Švarcová, Eva Kočí, Petr Bezdička, Silvia Garrappa, Libor Kobera, Jiří Plocek, Jiří Brus, Martin Šťastný, and David Hradil. Uncovering lead formate crystallization in oil-based paintings. *Dalton Transactions*, 49(16):5044–5054, 2020. doi: 10.1039/D0DT00327A.
